# A rigorous *in silico* genomic interrogation at 1p13.3 reveals 16 autosomal dominant candidate genes in syndromic neurodevelopmental disorders

**DOI:** 10.3389/fnmol.2022.979061

**Published:** 2022-10-06

**Authors:** Afif Ben-Mahmoud, Kyung Ran Jun, Vijay Gupta, Pinang Shastri, Alberto de la Fuente, Yongsoo Park, Kyung Chul Shin, Chong Ae Kim, Aparecido Divino da Cruz, Irene Plaza Pinto, Lysa Bernardes Minasi, Alex Silva da Cruz, Laurence Faivre, Patrick Callier, Caroline Racine, Lawrence C. Layman, Il-Keun Kong, Cheol-Hee Kim, Woo-Yang Kim, Hyung-Goo Kim

**Affiliations:** ^1^Neurological Disorders Research Center, Qatar Biomedical Research Institute, Hamad Bin Khalifa University, Doha, Qatar; ^2^Department of Laboratory Medicine, Inje University Haeundae Paik Hospital, Busan, South Korea; ^3^Department of Cardiovascular Medicine, Cape Fear Valley Medical Center, Fayetteville, NC, United States; ^4^Diabetes Research Center, Qatar Biomedical Research Institute, Hamad Bin Khalifa University, Doha, Qatar; ^5^Faculdade de Medicina, Unidade de Genética do Instituto da Criança – Hospital das Clínicas HCFMUSP, Universidade de São Paulo, São Paulo, Brazil; ^6^School of Medical and Life Sciences, Genetics Master Program, Replicon Research Group, Pontifical Catholic University of Goiás, Goiânia, Brazil; ^7^Genetics Master Program, Replicon Research Nucleus, School of Agrarian and Biological Sciences, Pontifical Catholic University of Goias, Goiás, Brazil; ^8^Inserm UMR 1231 GAD, Genetics of Developmental Disorders, Université de Bourgogne-Franche Comté, Dijon, France; ^9^Centre de Référence Anomalies du Développement et Syndromes Malformatifs, Hôpital d’Enfants, Dijon, France; ^10^UMR 1231 GAD, Inserm – Université Bourgogne-Franche Comté, Dijon, France; ^11^Section of Reproductive Endocrinology, Infertility and Genetics, Department of Obstetrics and Gynecology, Augusta University, Augusta, GA, United States; ^12^Department of Neuroscience and Regenerative Medicine, Augusta University, Augusta, GA, United States; ^13^Department of Animal Science, Division of Applied Life Science (BK21 Four), Gyeongsang National University, Jinju, South Korea; ^14^Department of Biology, Chungnam National University, Daejeon, South Korea; ^15^Department of Biological Sciences, Kent State University, Kent, OH, United States

**Keywords:** 1p13.3 deletion, intellectual disability, autism, *VAV3*, *WDR47*, *ELAPOR1*, *GSTM5*, *LRIF1*

## Abstract

Genome-wide chromosomal microarray is extensively used to detect copy number variations (CNVs), which can diagnose microdeletion and microduplication syndromes. These small unbalanced chromosomal structural rearrangements ranging from 1 kb to 10 Mb comprise up to 15% of human mutations leading to monogenic or contiguous genomic disorders. Albeit rare, CNVs at 1p13.3 cause a variety of neurodevelopmental disorders (NDDs) including development delay (DD), intellectual disability (ID), autism, epilepsy, and craniofacial anomalies (CFA). Most of the 1p13.3 CNV cases reported in the pre-microarray era encompassed a large number of genes and lacked the demarcating genomic coordinates, hampering the discovery of positional candidate genes within the boundaries. In this study, we present four subjects with 1p13.3 microdeletions displaying DD, ID, autism, epilepsy, and CFA. *In silico* comparative genomic mapping with three previously reported subjects with CNVs and 22 unreported DECIPHER CNV cases has resulted in the identification of four different sub-genomic loci harboring five positional candidate genes for DD, ID, and CFA at 1p13.3. Most of these genes have pathogenic variants reported, and their interacting genes are involved in NDDs. RT-qPCR in various human tissues revealed a high expression pattern in the brain and fetal brain, supporting their functional roles in NDDs. Interrogation of variant databases and interacting protein partners led to the identification of another set of 11 potential candidate genes, which might have been dysregulated by the position effect of these CNVs at 1p13.3. Our studies define 1p13.3 as a genomic region harboring 16 NDD candidate genes and underscore the critical roles of small CNVs in *in silico* comparative genomic mapping for disease gene discovery. Our candidate genes will help accelerate the isolation of pathogenic heterozygous variants from exome/genome sequencing (ES/GS) databases.

## Introduction

Chromosomal deletions and duplications, called copy number variations (CNVs), are unbalanced chromosomal structural abnormalities that may result in genomic disorders, the manifestations of which are frequently syndromic neurodevelopmental disorders (NDDs) (Lee et al., 2015). With its genomic length of 249 Mb, chromosome 1 is the largest human chromosome and contains about 2,000 human genes comprising about 9% of the total human genes (Murphy et al., 2003). A large number of genes on this chromosome are involved in mental and physical development, and thus, large heterozygous deletions or duplications can cause congenital abnormalities or pregnancy loss (Chen et al., 2017).

Subjects with CNVs within the proximal short arm of chromosome 1, specifically in the 1p13.3 region, are rare, and only seven cases have been reported so far (Tabata et al., 1991; Mattia et al., 1992; Lo et al., 1998; Utkus et al., 1999; Bisgaard et al., 2007; van Kuilenburg et al., 2009; Piccione et al., 2010). Importantly, these seven subjects share an overlapping phenotype of developmental delay (DD), intellectual disability (ID), epilepsy, and craniofacial anomalies (CFA) (Cases 1–7), suggesting the presence of syndromic NDD disease genes within 1p13.3. Four of them (Cases 4–7) are from the pre-comparative genomic hybridization (CGH) era without demarcating coordinates and thus not very informative (Table 1).

**TABLE 1:** Clinical features of eleven CNV subjects with 1p13.3 microdeletion/microduplication. Our four CNV carriers 1–4 are indicated here as Subjects 1–4.

Subjects ID	Subject 1 DGDP030	Subject 2	Subject 3	Subject 4	Case 1 Bisgaard et al., 2007	Case 2 van Kuilenburg et al., 2009	Case 3 Piccione et al., 2010	Case 4 Tabata et al., 1991	Case 5 Mattia et al., 1992	Case 6 Lo et al., 1998	Case 7 Utkus et al., 1999
Genomic coordinates [hg19]	chr1:107,240, 429-110,671,860	chr1:108,726, 456-108,853,796	chr1:108,729, 365-108,853,796	chr1:109,878, 638-110,200,728	chr1:104,092, 487-116,035,695	chr1:95,558,073-109,584,850 (personal communication, May 19, 2022)	chr1:102,540, 642-112,979,474	Unknown	Unknown	Unknown	Unknown
Cytogenetic Band	del(1)(p13.3)	del(1)(p13.3)	del(1)(p13.3)	del(1) (p13.3)*mat*	del(1) (p13.3p21.1)	del(1) (p13.3p21.3)	dup(1) (p21.2p13.2)	del(1) (p13.3p22.3)	del(1) (p13.3p22.3)	dup(1) (p13.1p22.1)	46,XY,der(6),ins(6;1) (q25;p13.3p22.1)*pat*
CNV	Deletion	Deletion	Deletion	Deletion	Deletion	Deletion	Duplication	Deletion	Deletion	Duplication	Duplication
Size (Mb)	3.4	0.13	0.12	0.322	11.9	14	10.44	¿18.8	>*18*.*8*	¿21.4	>*12*.*4*
Inheritance	Adopted	*de novo*	*de novo*	Maternal with the same phenotype	*de novo*	*de novo*	*de novo*	*de novo*	*de novo*	*de novo*	Paternal insertional translocation 46,XY,ins(6;1) (q25;p13.3p22.1)mat
Method of detection	FISH and array-CGH	Array-CGH	Array-CGH	Array-CGH	Array-CGH	FISH and array-CGH	Array-CGH	karyotyping	karyotyping	karyotyping	GTG banding and FISH
Age	44 years	11 years 6 months	6 years	22 years	13 years	3.5 years Patient 5	17 years	Died at 7 months	2 years	8 years	3 years
Sex	F	M	F	F	F	M	M	F	M	M	M
Ethnicity	Asian	European	European	European	N/A	European	N/A	N/A	European	Chinese	N/A
Developmental delay	+ Teeth did not appear until 20 months of age, severely developmentally delayed	+ Sat independently at around 12 months, walked at 24 months	+ Sat independently at around 10 months and walked at 16 months, no sucking reflex as new born	+ Walked at 18 months	+ No toilet trained, at 4.5 years, she was able to sit by herself and to walk for a short distance with support, failed to thrive until she received a gastrostomy at the age of eight years, first signs of puberty at the age of 12 years	+ Psychomotor impairment, feeding difficulties	+ Psychomotor retardation, walking independently occurred when he was one year old	+ Growth and psychomotor retardation	+ Early delays in motor skills	+	+ Sat at 9 months and walked independently at 17 months of age
Speech/ language delay	+ Talked to some extent until 3 year, only a few words	+ Spoke his first words at 36 months and was not speaking full sentences until 11 years of age	N/A	N/A	+ Never learned language	N/A	+ Language retardation	N/A	+ Delay	N/A	+ No speech
Intellectual disability	+	+	+	+	+ Severe	+	+ IQ -64	N/A Died at seven months	N/A	+	+
Learning disability	+	+ Severe learning difficulties	N/A	N/A	N/A	N/A	N/A	N/A	N/A	N/A	N/A
Autism	+ Did not make eye contact, autistic	_	_	_	N/A	+	+ Difficulty with establishing relationships	N/A	N/A	N/A	N/A
Cranial anomalies	+ Asymmetrical skull with smaller right parietal area	_	_	+ Microcephaly OFC < -2DS	+ Microcephaly head circumference was 49.5 cm (<3rd centile)	+ Macrocephaly, epiphyseal dysplasia of the femoral head, frontal bossing, prominent forehead	+ Microcephaly (25th–50th centile)	_	+ At birth macrocephaly, OFC (90th centile) and at 22 months normocephaly, frontal bossing with ridged metopic suture, prominent occiput	+ Microcephaly (<3rd centile)	+ Microcephaly at 38 weeks and macrocephaly at three years age, wide prominent forehead with high frontal hairline, flat occiput, one occipital hair whorl in the midline
Facial dysmorphism	+ Ptosis, low-set ears, unfolded ears, and high arched palate	+ Bulbous nasal tip, epicanthal folds, downslanted palpebral fissure, low anterior hairline, long philtrum, wide nasal bridge, thin upper lip vermilion, long ears, micrognathi, and hypertelorism	+ Epicanthal folds, downslanted palpebral fissure, long philtrum, wide nasal bridge, hypertelorism, and micrognathia	+ Long face, epicanthus, prominent nose, posteriorly rotated ears	+ The extremities were short compared with the body, the shoulders were narrow and the neck was webbed, the hairlines, front and back were low, and the hair and eyebrows were thick and dark, the ears were low set, the ear lobes broad and the crus helix on the left ear was prominent, a broad nasal bridge and tip, anteverted nares, a prominent premaxillary region and an open mouth with an everted lower lip, her palate was high arched and there was space between her upper incisors	+ Hypertelorism, downward slanted palpebral fissures, low nasal bridge, full nasal tip, anteverted nares, long and prominent philtrum, open mouth appearance, everted lower vermillion, a highly arched palate and large lobules, large anterior fontanel, epicanthal folds, broad flat root of nose	+ Small receding chin, prominent nasal bridge, flat nose, short philtrum, midface hypoplasia	+ Upper and lower eyelids connected to each other by a string-like epithelium, low hairline, epicanthal folds, saddle nose with a broad, flat root, micrognathia, short neck, high-arched palate	+ Large anterior fontanelle, forehead nevus flammeus, low set ears with overlapping helices, bilateral ptosis (right greater than left), deeply set eyes, downward slanting palpebral fissures, arched eyebrows, long eyelashes, prominent nasal bridge and nasal tip, thick columella, and mild micrognathia, moderate right ptosis, short neck	+ Flare of the lateral eyebrows, eversion of the lateral part of lower eyelids, long eyelashes, epicanthic folds, blue sclerae, flat nose with short columella, prominent ears, mild micrognathia, bilateral ptosis	+ Hypertelorism, horizontal palpebral fissures, small lower incisors with diastema, narrow and high palate, large and prominent, but not misshapen ears
Epilepsy /seizures /spasms	+ Suffered febrile seizures at 18 months, possible myoclonic seizures,	_	+ Episode of seizure and her MRI indicated a small arachnoid cyst in the posterior fossa	_	+ Epilepsy	+	N/A	+ Several episodes of generalized tonic clonic convulsions without high fever	N/A	+ Several tonic seizures at age 7 years	N/A
Cardiac anomalies	_	N/A	N/A	N/A	N/A	N/A	_	+ Extreme tetralogy of Fallot	_	_	+ Cardiac sonography disclosed a chorda running through the left ventricle
Short stature	+	+	_	+	+ <5th centile	+ 50th centile height	N/A	N/A	_	_	_
Hearing loss	+	N/A	N/A	N/A	N/A	N/A	N/A	N/A	N/A	+ Hearing deficit on the left side	N/A
Behavioral problems	+ Childhood schizophrenia, hallucinations, obsessive compulsive, wide mood swings, being in a room with people but did not make much effort to interact, complete emotional breakdown and increased violent behavior, strange night terror episodes	+ Deficits in attention and executive functions	_	+ Anxiety	+	+ Psychomotor retardation	+ Stereotyped movements finger snapping and repeated mannerism with obsessive–compulsive behavior	N/A	N/A	N/A	+ Constantly moving around
Hand/finger /feet/toe anomalies	+ Walk with arched feet, cramps in her legs and arms, held her hands at odd angles and held objects with her fingertips, jerky gait	N/A	N/A	+ Short fingers brachydactyly	+ Valgus feet, a groove between the first and second toes, and minimal 2–3 syndactyly on the right foot, hypermobility	+ Nails were short and thin, tapering fingers (Figure 1)	+ Arachnodactyly of the fingers, fingers and toes with joint hyperlaxity	+ Bilateral pes equinovarus, fifth toes that overlapped the fourth toes bilaterally, a deep fissure between the first and second toes bilaterally, and abnormal flexions of fingers and toes, bilateral flexions of the radiocarpal, first metacarpophalangeal, hallucal interphalangealjoints, overextension of the first metatarsophalangeal joint	+ Arched feet, joint laxity and a high arched right foot, dermal ridge patterns showed a central whorl on the right thumb, second, and fifth digits, a biradial loop on the left thumb, ulnar loops on the right fourth and left	+ Persistent fetal finger pads, mild joint laxity, and mild cutaneous syndactyly between all fingers and toes	+ Flat arches of the feet, and irregular position of toes with tibial deviation of the second toes, gait was peculiar with outward rotation of the feet, small hands, brachydactyly, clinodactyly of 5th fingers
Muscle disorder	+ Mild fiber atrophy and random variation in muscle fiber size, motor milestones were of poor quality and out of the usual order	_	_	_	+ hypotonia, diplegia	+ Hypertonia and hyperreflexia changed into severe hypotonia and areflexia	N/A	N/A	+ Mild hypotonia, most notably in the upper limbs	N/A	+ Hypotonia
Eye disorder	+ Strabismus, astigmatism	N/A	N/A	N/A	+ Coloboma of both irides, central vision impairment	+ Myopia, astigmatism and nystagmus	+ Astigmatism	N/A	+ Ptosis with deeply set eyes	+ Long eye-lashes, eversion of the lateral part of lower eye-lids, bilateral ptosis	+ Mild exophthalmos
Other	Mild anemia, recurring petechial rash, kyphosis, lordosis		Loose skin	Mother and oldest brother show similar neurodevelop- mental phenotypes	Respiratory infections	Respiratory problems and eruption of his dentition was delayed,		Prominent xiphisternum, wide-spaced nipples, cyanosis and multiple congenital anomalies, hypoxia and metabolic acidosis	High-pitched cry showed mild gastroesopha geal reflux and an asymptomatic malrotation with a left-sided appendix	Kabuki make-up syndrome, episode of gastroenteritis at 5 months, recurrent otitis media with residual high-tone	Umbilical hernia, hypoplastic nipples, sacral dimple

**FIGURE 1:**
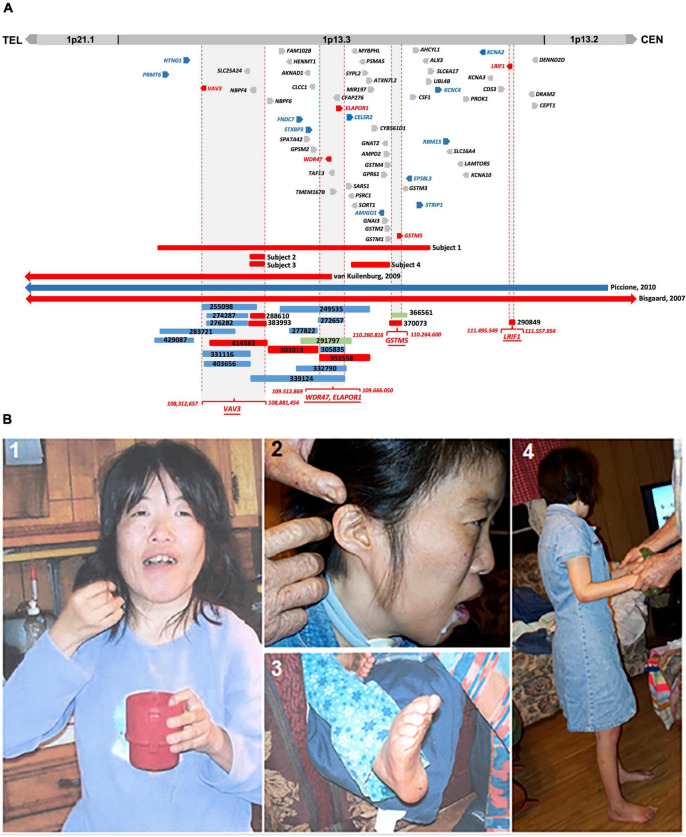
**(A)** Comparative CNV mapping of subjects with CNVs at 1p13.3. Deletions are represented by red bars and duplications are represented by blue bars. CNV cases from the DECIPHER database are denoted by a six-digit reference number. The sizes of the deleted and duplicated regions from Bisgaard et al. (2007) (Case 1), van Kuilenburg et al. (2009) (Case 2), Piccione et al. (2010) (Case 3), and 22 DECIPHER cases are presented relative to our four subjects. Vertical dotted red lines flanking gray background represent the refined four candidate gene loci; 59 genes located within the 4.6 Mb genomic region at 1p13.3 are depicted either in red for positional candidate genes, blue for functional candidate genes, or gray for the remaining genes. **(B)** Phenotypic features of Subject 1. **(1)** Picture shows facial dysmorphism, asymmetrical head, and small hands; **(2)** Low set, small, and unfolded ears; **(3)** arched feet; and **(4)** full body picture showing short stature.

These three published subjects (Cases 1–3) have been featured in Figure 1A, whereas the other four subjects (Cases 4–7) from pre-microarray era with no demarcation are not. Genomic coordinates of Case 2 were solicited from the author, and “tapering fingers” of Case 2 were deduced from Figure 1 of the article. N/A denotes not available, while (-) represents absence of the corresponding phenotype. Not tested; some cases are before the microarray era and without genomic coordinates.

**FIGURE 2:**
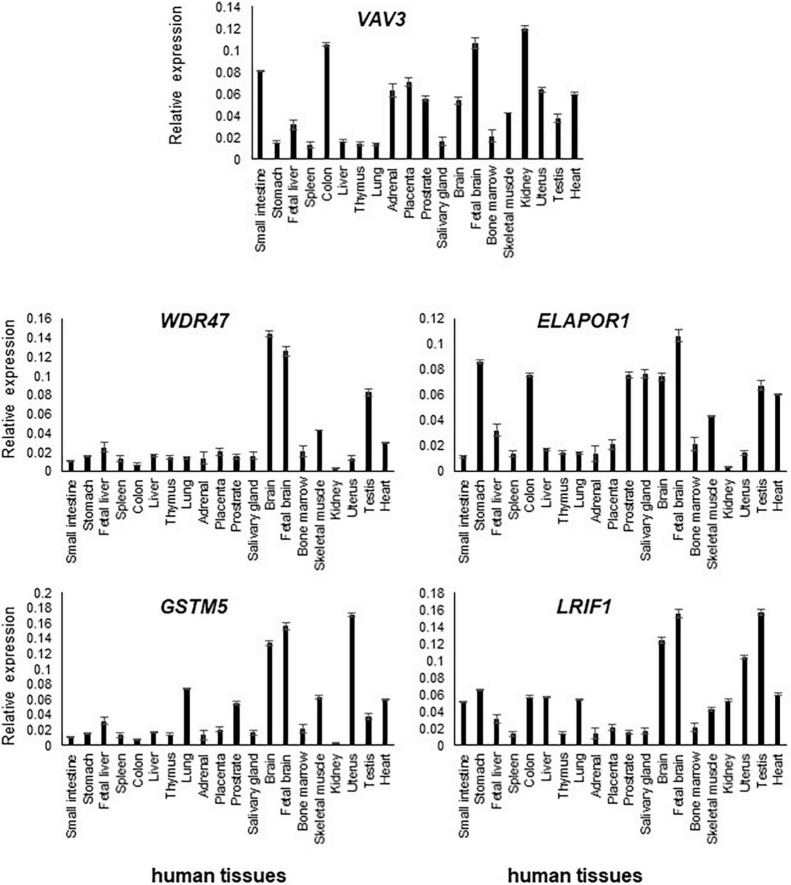
Transcript levels of *VAV3, ELAPOR1, WDR47, GSTM5*, and *LRIF1* in various human tissues were determined by RT-qPCR. Relative high expression of them was detected in the adult and fetal brain compared to other tissues.

**FIGURE 3:**
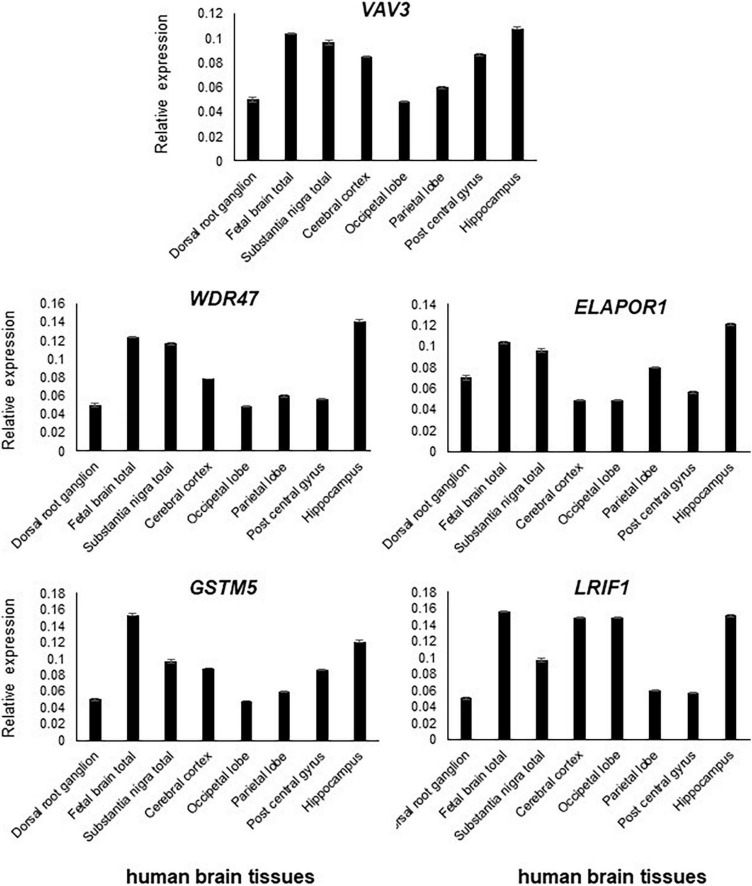
All five genes, namely, *VAV3, WDR47, ELAPOR1, GSTM5*, and *LRIF1*, are expressed in the various parts of the brain with varying levels.

In this study, we describe the clinical phenotype of four subjects having microdeletions of different sizes at 1p13.3 with an overlapping phenotype of DD, ID, autism, epilepsy, and CFA. *In silico* comparative genomic mapping of these four subjects (Subjects 1–4 on Figure 1A and Table 1) with three reported demarcated deletions and duplication (Cases 1-3 on Table 1 and Figure 1A) alongside twenty-two informative DECIPHER cases^1^, version 11.14) (Firth et al., 2009; Figure 1A and Supplementary Table 3) has allowed us to narrow down the 1p13.3 genomic region harboring approximately 59 genes to four sub-genomic segments that encompass five positional candidate genes for syndromic NDDs at 1p13.3 (Figure 1A and Table 2). Upon analyzing various human disease databases including Human Gene Mutation Database (HGMD, ^2^ version Professional 2022.2), MGI (Mouse Genome Informatics^3^, version 6.21), BioGrid^4^ (version 4.4.212), and STRING^5^ (version 11.5), we found that these five candidate genes have either NDD-associated nucleotide variants reported in them or their interactors, and a behavioral phenotype was shown in KO mice (Table 2). A high RNA expression pattern of these five positional candidate genes in human adult and fetal brain further supported this postulate (Figures 2, 3). Individual interrogation of the 54 remaining genes identified another set of 11 candidate genes. Their candidacy was substantiated by sporadic variants reported in them and their interactors, and their physical interaction with known NDD genes based on BioGrid and STRING, as well as KO mice phenotype. (Supplementary Table 1). Disease Gene Network Analysis (DisGeNET^6^, version 7.0) and HPO (Human Phenotype Ontology^7^, version 1.7.16) provided additional substantiation of the candidacy of our candidate genes in NDDs.

**TABLE 2:** Five positional candidate genes for syndromic NDDs identified by *in silico* CNV mapping at 1p13.3.

No.	Gene candidate & MIM	Variants in human and KO mice with NDDs phenotype	Protein interactors (references for interaction and variants in human with NDDs)
1	*VAV3* (VAV guanine nucleotide exchange factor 3) (605541)	1. Autism: *de novo* c.2305C > G (NM_006113.5); p.P769A (NP_006104.4) (Neale et al., 2012; Turner et al., 2019) 2. Autism spectrum disorder: *de novo* c.1009delC (NM_006113.5); p.L337Sfs*18 (NP_006104.4) (Iossifov et al., 2014; Li et al., 2017) 3. Autism spectrum disorder, increased risk of: *de novo* c.758A > G (NM_006113.5); p.H253R (NP_006104.4) (Lim et al., 2017) 4. Developmental disorder: *de novo* c.1897C > T (NM_006113.5); p.H633Y (NP_006104.4) (Turner et al., 2019) 5. Schizophrenia: *de novo* c.2222A > G (NM_006113.5); p.E741G (NP_006104.4) (Aleksic et al., 2013) 6. In mice, knocking out the *Vav3* gene produces broad physiological changes due to significant issues with sympathetic nervous system modulation (SNS). This is because Vav3 is involved in the formation of appropriate inhibitory GABAergic circuitry between the caudal (CVLM) and rostral (RVLM) ventrolateral medullas in the brainstem (Sauzeau et al., 2010; Rodriguez-Fdez et al., 2021) 7. In the postnatal stage, *Vav3* mice display significant motor coordination and gaiting impairments. These findings suggest that Vav3 function is important for the cerebellum’s timely development (Quevedo et al., 2010)	1. *RAC1* (Movilla and Bustelo, 1999): developmental delay, brain malformation, microcephaly (Reijnders et al., 2017), intellectual disability (Lelieveld et al., 2016; Reijnders et al., 2017) 2. *EGFR* (Erdem-Eraslan et al., 2015): autism spectrum disorder (Iossifov et al., 2014; Kosmicki et al., 2017) 3. *IGF1R* (Zeng et al., 2000): short stature, intellectual disability, microcephaly, hypotonia, lack of speech (Uehara et al., 2016) 4. *ZRANB1* (Luck et al., 2020): developmental disorder (Deciphering Developmental Disorders, 2017; Kosmicki et al., 2017; Turner et al., 2019) 5. *TARDBP* (Muller et al., 2020): amyotrophic lateral sclerosis (Kabashi et al., 2008; Sreedharan et al., 2008)
2	*WDR47* (WD repeat-containing protein 47) (615734)	1. Autism spectrum disorder: *de novo* c.991_992delAT (NM_014969.6); p.I331Ffs*16 (NP_055784.3) (Callaghan et al., 2019) 2. The neuroanatomical deficits associated with *Wdr47* KO cause hyperactivity and inappropriate sensory motor gating in both male and female mice (Kannan et al., 2017). 3. The overall brain size of *Wdr47^tm1a/tm1a^* mice was reduced, indicating initial microcephaly that worsened postnatally (Kannan et al., 2017). 4. Mutations in 27 WDR genes (∼9 %) have been linked to brain disorders especially intellectual disability related to corpus callosum defects (Kannan et al., 2017).	1. *TCF4* (Li et al., 2015): Pitt-Hopkins syndrome with neurodevelopmental phenotypes (Amiel et al., 2007; Zweier et al., 2007), intellectual disability (Mary et al., 2018) 2. *ANGPTL4* (Huttlin et al., 2021): autism spectrum disorder (Iossifov et al., 2014), developmental disorder (Turner et al., 2019) 3. *DYRK1A* (Guard et al., 2019): autism spectrum disorder (Guo et al., 2018), microcephaly, intellectual disability, speech impairment and distinct facial features (Ji et al., 2015) 4. *RAB11FIP5* (Huttlin et al., 2021): autism spectrum disorder (Roohi et al., 2008; Matsunami et al., 2014), autism (Yuen et al., 2016) 5. *STRN* (Huttlin et al., 2021): autism spectrum disorder (Kosmicki et al., 2017; Lim et al., 2017; Satterstrom et al., 2020), bipolar disorder (Toma et al., 2020) 6. *KRAS* (Martin et al., 2017): Noonan syndrome (Schubbert et al., 2006; Zenker et al., 2007; Leventopoulos et al., 2010; Turro et al., 2020; Ando et al., 2021), intellectual disability and multiple congenital abnormalities (Vergult et al., 2014), neurodevelopmental disorder (Popp et al., 2017)
3	*ELAPOR1* (Endosome-lysosome-associated apoptosis and autophagy regulator 1) (611298)	1. Developmental disorder: *de novo* c.2482G > A (NM_001267048.2); p.A828T (NP_001253977.2) / *de novo* c.2653G > T (NM_001267048.2); p.D885Y (NP_001253977.2) (Turner et al., 2019) 2. Autism spectrum disorder: *de novo* c.2341G > A (NM_001267048.2); p.V781I (NP_001253977.2) (Iossifov et al., 2014) 3. Autism: *de novo* c.2341G > A (NM_001267048.2); p.V781I (NP_001253977.2) (Turner et al., 2019) 4. KO mouse phenotypes associated with decreased exploration in new environment: http://www.informatics.jax.org/diseasePortal/popup?isPhenotype=true&markerID=MGI:1923930&header=behavior/neurological	1. *OSBPL3* (Huttlin et al., 2021): autism (Turner et al., 2019) 2. *DDX58* (Wu et al., 2020): autism spectrum disorder (Iossifov et al., 2014; Lim et al., 2017), autism (Turner et al., 2019) 3. *ADAM15* (Huttlin et al., 2021): autism spectrum disorder (Lim et al., 2017) 4. *ATP13A1* (Huttlin et al., 2021): developmental disorder (Tran Mau-Them et al., 2020), intellectual disability (Anazi et al., 2017; Kahrizi et al., 2019) 5. *ATP6AP2* (Huttlin et al., 2021): mental retardation and epilepsy (Ramser et al., 2005; Al-Nabhani et al., 2018), developmental disorder (Turner et al., 2019), intellectual disability, epilepsy and parkinsonism (Gupta et al., 2015), mental retardation (Buysse et al., 2009), neurodevelopmental disorder (Zhang et al., 2021) 6. *CNTNAP3* (Huttlin et al., 2021): autism spectrum disorder (An et al., 2014; Le et al., 2019), simplex autism (Turner et al., 2017)
4	*GSTM5* (Glutathione S-Transferase Mu 5) (138385)	1. GSTM5 belongs to the glutathione S-transferase enzyme family. GSTM5 is located in the brain and metabolizes a wide range of substances, both exogenous and endogenous (Eaton and Bammler, 1999) 2. GSTs (Glutathione S-transferases), which are involved in the glutathione metabolism pathway and include GstA3, Gstm1, Gstm5, Gstm3, Gstk1, and Gstp1, have been identified as risk factors for Alzheimer’s disease (Lin et al., 2017) 3. Polymorphisms in *GSTM5* predicted microRNA binding sites were associated with Parkinson’s disease diagnosis age (Searles Nielsen et al., 2013) 4. KO mouse phenotypes associated with decreased exploration in new environment: http://www.informatics.jax.org/diseasePortal/genoCluster/view/44861	1. *ERLIN2* (Huttlin et al., 2021): spastic paraplegia (Morais et al., 2017; D’Amore et al., 2018; Rydning et al., 2018; Travaglini et al., 2018; Wright et al., 2018; Park et al., 2020), intellectual disability(Najmabadi et al., 2011; Hu et al., 2019), motor dysfunction and joint contractures (Yildirim et al., 2011) 2. *ARFGAP1* (Huttlin et al., 2021) : autism spectrum disorder (Satterstrom et al., 2020) 3. *AGPAT1* (Huttlin et al., 2021) : autism spectrum disorder (Callaghan et al., 2019) 4. *CDC42* (Silva et al., 2019): intellectual disability, brain malformations, and facial dysmorphism (Martinelli et al., 2018), Noonan-like syndrome (Martinelli et al., 2018), epileptic encephalopathy with infantile spasms (Helbig et al., 2016; Martinelli et al., 2018), intellectual disability, brain malformations, and platelet anomalies (Martinelli et al., 2018), facial dysmorphism, neurodevelopmental delay (Szczawinska-Poplonyk et al., 2020), schizophrenia (Gilks et al., 2012) 5. *GPKOW* (Luck et al., 2020): autism spectrum disorder (Al-Mubarak et al., 2017), microcephaly and intrauterine growth restriction (Carroll et al., 2017)
5	*LRIF1* (Ligand-dependent nuclear receptor-interacting factor 1) (1615354)	1. Developmental disorder: *de novo* c.1151_1152delCT (NM_018372.4); p.S384Cfs*4 (NP_060842.3) (Turner et al., 2019) 2. LRIF1 is required for accurate chromosome segregation in mitosis (Akram et al., 2018) and genes encoding mitotic regulators are growingly linked to neurodevelopmental diseases (Degrassi et al., 2019).	1. *PLEKHA4* (Shami Shah et al., 2019): autism spectrum disorder (Iossifov et al., 2014; Lim et al., 2017), autism (Hashimoto et al., 2016; Turner et al., 2019) 2. *CBX5* (Luck et al., 2020): autism spectrum disorder (Satterstrom et al., 2020), developmental disorder (Deciphering Developmental Disorders, 2017), schizophrenia (Gulsuner et al., 2013) 3. *RARA* (Li et al., 2007): autism spectrum disorder (Iossifov et al., 2014) 4. *AHDC1* (Marcon et al., 2014): syndromic expressive language delay, hypotonia & sleep apnoea (Xia et al., 2014), Xia-Gibbs syndrome (Garcia-Acero and Acosta, 2017; Jiang et al., 2018; Wang et al., 2020; Faergeman et al., 2021), neurodevelopmental disorder (Wang et al., 2020), intellectual disability and developmental delay (Yang et al., 2015; Pekeles et al., 2019), autism spectrum disorder (Iossifov et al., 2014; Kosmicki et al., 2017; Lim et al., 2017), moderate intellectual disability, speech delay, macrocephaly, facial dysmorphism, cleft palate, hypertelorism & macrocrania (Bowling et al., 2017; Jiang et al., 2018) 5. *PQBP1* (Stelzl et al., 2005): mental retardation (Kalscheuer et al., 2003; Lenski et al., 2004; Jensen et al., 2011; Rahman et al., 2019), intellectual disability (Redin et al., 2014; Grozeva et al., 2015; Hu et al., 2016; Abdel-Salam et al., 2018), microcephaly (Shaheen et al., 2019) 6. *SETD1B* (Goehler et al., 2004): intellectual disability and seizures (Brunet et al., 2021; Roston et al., 2021), Intellectual disability, developmental delay, epilepsy, language disorder, autism, facial dysmorphism (Palumbo et al., 2015; Faundes et al., 2018; Hiraide et al., 2018, 2019, 2021), autism spectrum disorder (Iossifov et al., 2014; Satterstrom et al., 2020), Developmental and epileptic encephalopathy (Takata et al., 2019), schizophrenia (Wang et al., 2015) 7. *CDC42* (Stelzl et al., 2005): intellectual disability, brain malformations, and facial dysmorphism (Martinelli et al., 2018), Noonan-like syndrome (Martinelli et al., 2018), epileptic encephalopathy with infantile spasms (Helbig et al., 2016; Martinelli et al., 2018), intellectual disability, brain malformations, and platelet anomalies (Martinelli et al., 2018), facial dysmorphism, neurodevelopmental delay (Szczawinska-Poplonyk et al., 2020), schizophrenia (Gilks et al., 2012)
			8. *ATP1B1* (Stelzl et al., 2005): intellectual disability & self-mutilation (Liu et al., 2015), autism(Iossifov et al., 2012; Iossifov et al., 2014; Uddin et al., 2014), autism spectrum disorder (Lim et al., 2017; Turner et al., 2019), intellectual disability (Liu et al., 2015), schizophrenia (Purcell et al., 2014) 9. *CHD3* (Goehler et al., 2004): neurodevelopmental syndrome with macrocephaly, impaired speech and language (Snijders Blok et al., 2019), developmental disorder (Deciphering Developmental Disorders, 2017; Kosmicki et al., 2017; Snijders Blok et al., 2019; Turner et al., 2019), autism (Iossifov et al., 2014; Yuen et al., 2016; Kosmicki et al., 2017; Turner et al., 2019), autism spectrum disorder (Wang et al., 2016; Lim et al., 2017; Cappi et al., 2020; Satterstrom et al., 2020) 10. *CHD4* (Marcon et al., 2014): Sifrim-Hitz-Weiss syndrome (Weiss et al., 2020), neurodevelopmental disorder (Trinh et al., 2019), intellectual disability (Weiss et al., 2016, 2020), developmental disorder (Deciphering Developmental Disorders, 2017; Turner et al., 2019; Weiss et al., 2020), autism spectrum disorder (Iossifov et al., 2014; Wang et al., 2016; Kosmicki et al., 2017), intellectual disability, macrocephaly, hyperlaxity of finger joints and hearing loss (Monroe et al., 2016; Weiss et al., 2016), schizophrenia (Girard et al., 2011; Li et al., 2016)

The candidacy of these genes was justified by their genomic positions in CNV mapping, sporadic variants reported in them and their interactors in NDDs, their physical interaction with known neurodevelopmental genes, and KO mice phenotype based on HGMD, BioGrid, STRING, and MGI. Due to a large number of interacting genes, only a limited number of them in NDD were described. KO mice data with neurobehavioral phenotype are also mentioned wherever available.

## Results

### Microarray

Microarray analysis carried out on genomic DNA from Subject 1 revealed a 3.4 Mb heterozygous deletion at 1p13.3 (chr1: 107,240,429-110,671,860, [hg19]) and its inheritance was not determined. Additional microarray performed on Subject 2, Subject 3, and Subject 4 revealed a 130 kb del(1)(p13.3) arr[hg19](chr1: 108,726,456-108,853,796)x1*dn*, 120 kb del(1)(p13.3) arr[hg19](chr1; 108,729,365-108,853,796)x1*dn*, and 322 kb del(1)(p13.3) arr[hg19](chr1: 109,878,638-110,200,728)x1*mat*, respectively (Table 1 and Figure 1A). Subject 4 has inherited the deletion from her mother with the same phenotype.

### Identification of five positional candidate genes by *in silico* comparative genomic mapping

We compared the clinical phenotypes and flanking genomic boundaries displayed in our four subjects (Subjects 1–4) with three previously reported CNV cases encompassing 1p13.3 (Cases 1–3) (Bisgaard et al., 2007; van Kuilenburg et al., 2009; Piccione et al., 2010) (Table 1 and Figure 1A) as well as 22 CNVs with NDDs from a total of 82 cases in the DECIPHER database, based on the criteria of less than 1 Mb in size (Supplementary Table 3). *In silico* comparative CNV mapping resulted in four refined candidate gene loci, where the maximal number of CNVs are overlapped (Figure 1A), and identified five positional candidate genes (Table 2). Loci one, three, and four have a single candidate gene, *VAV3, GSTM5*, and *LRIF1*, respectively, whereas locus two has two positional candidate genes, *WDR47* and *ELAPOR1*.

*VAV3* (VAV guanine nucleotide exchange factor 3, MIM 605541) from locus 1 is a member of the VAV family of guanine nucleotide exchange factors for Rho and Rac GTPases. The VAV family is a group of signal transduction molecules that are regulated by tyrosine phosphorylation and positioned downstream of protein tyrosine kinases. In the ventral medulla, *VAV3* is involved in cerebellum development and axon wiring processes. In the cerebellum, it is involved in Purkinje cell dendritogenesis and the migration and survival of granule cells (Quevedo et al., 2010). In the early postnatal stages of KO mice, the loss of *VAV3* causes motor coordination defects and abnormal gait (Quevedo et al., 2010). Haploinsufficiency of *VAV3* and other four genes (*NTNG1*, *LPPR4*, *GPSM2*, and *COL11A1*) was postulated to be contributing factors toward the psychomotor impairment and abnormal craniofacial features reported in a subject with a 14 Mb interstitial deletion encompassing 1p13.3 (van Kuilenburg et al., 2009). Interestingly, three *de novo* variants in *VAV3* were found in individuals with autism spectrum disorder (ASD) (Neale et al., 2012; Iossifov et al., 2014; Li et al., 2017; Lim et al., 2017; Turner et al., 2019), one with neurodevelopmental disorder (Turner et al., 2019), and one with schizophrenia (Aleksic et al., 2013; Table 2).

*SLC25A24* (solute carrier family 25 member 24, MIM 608744) is within candidate locus one, which also includes *NBPF4* (Figure 1A). Two recurrent missense mutations in *SLC25A24* have been reported in Gorlin-Chaudhry-Moss syndrome (GCMS) (Ehmke et al., 2017) and Fontaine syndrome (Writzl et al., 2017), which are progeroid syndromes associated with accelerated aging. Due to the extreme aging facial phenotype caused by reported heterozygous mutations, the low level of expression in the human brain, and the absence of sporadic variants in the subjects with DD or ASD from the literature, this gene was excluded from the positional candidate gene list.

*NBPF4* (neuroblastoma breakpoint family, member 4, MIM 613994) is a member of the neuroblastoma breakpoint family (NBPF) and contains the DUF1220 protein domain of approximately 65 amino acids in length. This domain has undergone unusually rapid and extensive duplications due to its repetitive structure during recent primate evolution. Chromosome 1 has 20 *NBPF* genes containing highly conserved DUF1220 domains and 13 of them are dispersed at 1q21 (Vandepoele et al., 2005; Diskin et al., 2009). Although DUF1220 sequences appear to exhibit a significant direct correlation with brain-size phenotypes in humans (Dumas et al., 2012), it was excluded due to no expression in the human brain and the absence of any reported genetic variants in NDDs.

Among the five genes, namely, *WDR47, TAF13, TMEM167B, CFAP276*, and *ELAPOR1*, located in the second locus, we found two positional candidate genes.

*WDR47* (WD repeat-containing protein 47, MIM 615734), sharing structural homology with lissencephaly gene *LIS1* (*PAFAH1B1*), participates in core microtubule-mediated processes, including neural stem cell proliferation, radial migration, and growth cone dynamics. KO mice lacking Wdr47 exhibited partial lethality, extensive fiber defects, microcephaly, thinner cortices, and abnormal sensory motor gait (Kannan et al., 2017). Kannan et al. speculated that mutations in this gene, particularly, truncating ones, would cause an embryonic lethal phenotype in humans, which would explain why no human mutations were identified. However, 2 years later, one frameshift variant in this gene was reported in a patient with ASD (Callaghan et al., 2019).

*TAF13* (TATA-box-binding-protein-associated factor 13, MIM 600774), associated with ID combined with microcephaly, was excluded due to its autosomal recessive pattern (Tawamie et al., 2017).

*TMEM167B* (transmembrane protein 167B, aka *C1orf119*) was not considered as a candidate because no genetic variants in this gene were reported in NDD subjects and it is not expressed at high levels in the human brain.

*CFAP276* (cilia and flagella-associated protein 276, aka *C1orf194*, MIM 618682) was excluded because two missense mutations cause dominant Charcott-Marie-Tooth disease (Sun et al., 2019); it is expressed at very low levels in the human brain, and no genetic variant was reported in NDD subjects.

Four genetic variants in *ELAPOR1* (endosome-lysosome associated apoptosis and autophagy regulator 1, aka *KIAA1324*, MIM 611298) in DD and ASD have been reported (Iossifov et al., 2014; Turner et al., 2019), and at least six of its interacting genes at the protein levels are strongly involved in diverse NDDs. Moreover, KO mice exhibit decreased exploration in a new environment (Table 2), suggesting *ELAPOR1* is likely a candidate gene (Figure 1A and Table 2).

The third candidate locus refined to 86 kb as depicted in Figure 1A implies *GSTM5* (glutathione S-transferase mu 5, MIM 138385), a member of the glutathione S-transferase (GST) family possibly involved in NDDs, because of variants found in interacting genes at the protein levels and KO mice showing decreased exploration in a new environment (Figure 1A and Table 2). Its paralog *GSTM1* null genotype in human carriers is more likely to develop ASD (Mandic-Maravic et al., 2021). GSTs are a group of enzymes that play a major role in the antioxidant defense mechanism by performing the inactivation of a large number of endogenous oxidative stress products. So far, a number of studies have suggested a link between oxidative stress and ASD (Mandic-Maravic et al., 2021). *GSTM5* has at least five interacting genes at the protein level, variants of which were reported in various NDD subjects (Table 2).

Finally, the fifth positional candidate gene *LRIF1* (ligand-dependent nuclear receptor-interacting factor 1, MIM 615354) is aligned near the centromeric region of 1p13.3. It overlaps with a 62 kb DECIPHER microdeletion case 290849 who presented with ID and behavioral abnormalities (Figure 1A and Supplementary Table 3). This variant was inherited from the father, whose phenotype is unknown. Importantly, however, *LRIF1* interacts with two genes among others at the protein level, namely, *PQBP1* (polyglutamine-binding protein 1, MIM 300463) and *CHD4* (chromodomain helicase DNA-binding protein 4, MIM 603277). Mutations in *PQBP1* are the cause of X-linked ID (Kalscheuer et al., 2003), while missense mutations in *CHD4* are linked to ID syndrome with distinctive dysmorphisms (Weiss et al., 2016). Furthermore, another interactor *ADHC1* (AT-hook DNA-binding motif-containing protein 1, MIM 615790) is involved in Xia-Gibbs syndrome with symptoms of ID, speech/motor delay, and facial dysmorphism (Xia et al., 2014), whereas interactor *CHD3* is associated with Snijders Blok-Campeau syndrome, an NDD exhibiting macrocephaly and speech/language delay (Snijders Blok et al., 2018). Missense variants of the interactor *CDC42* cause a diverse neurodevelopmental phenotype resembling Noonan syndrome (Martinelli et al., 2018), and the interactor *SETD1B* (aka *KMT2G*) is associated with the syndromic ID (Hiraide et al., 2018; Roston et al., 2021; Weerts et al., 2021; Table 2). We, therefore, suggest that *LRIF1* could be considered a strong candidate for NDD, and this single DECIPHER case underscores the possibility of finding a neurodevelopmental disease gene (Figure 1A).

We also checked the interacting proteins of these five positional candidate genes using STRING (Functional Protein Association Networks, string-db.org, version 11.5) and BIOGRID (Database of Protein, Chemical, and Genetic Interactions, thebiogrid.org, version 4.4) protein–protein interaction databases and found that all of them interact with proteins already known to be associated with various NDDs. This outlines the potential role played by the proteins encoded from these five positional candidate genes and their interacting protein partners (Table 2).

### Identification of 11 additional functional candidate genes possibly dysregulated by position effect

We have interrogated the remaining 54 genes at 1p13.3, some of which might be dysregulated by position effect even if they are not contained in the four candidate gene loci. The criteria for selecting additional candidate genes are 3-fold: (1) sporadic *de novo* genetic variants in them were reported in subjects with NDDs, (2) animal models substantiated their pathological roles in NDDs, and (3) sporadic *de novo* genetic variants in NDD subjects were reported in their interacting genes at the protein level. This approach has identified additional 11 candidate genes which might be dysregulated by position effect (Supplementary Table 1).

### Quantitative reverse transcription PCR (RT-qPCR)

Transcript levels of the five positional candidate genes were determined by RT-qPCR in various human tissues to assess their functional importance in phenotype-relevant tissues. The spatio-temporal regulation of gene expression leads to disparate expression patterns dependent on a variety of factors including the detection methods, which may not properly define the expression patterns. Differential expression patterns in multiple publicly available resources including GTEx Portal^8^ (version 8) and NCBI prompted us to use commercially available human RNA samples to measure the expression patterns through RT-qPCR experiment so that we would have a reference of expression of the genes of interest.

In comparison to other tissues, *VAV3, WDR47, ELAPOR1, GSTM5*, and *LRIF1* expressions were found relatively high in the adult brain and/or fetal brain (Figure 2). The high expression of these five genes in the human fetal brain suggests that they might be involved in an early neurodevelopmental phenotype such as DD when mutated. The variable expression pattern in tissues at different developmental stages underscores the significance of analyzing the fetal brain along with the adult brain.

All five genes (*VAV3, WDR47, ELAPOR1, GSTM5*, and *LRIF1*) were highly expressed in various subsections of the brain with differing levels (Figure 3).

### Disease-gene network analysis provides additional corroboration for NDD phenotype from candidate genes

**FIGURE 4:**
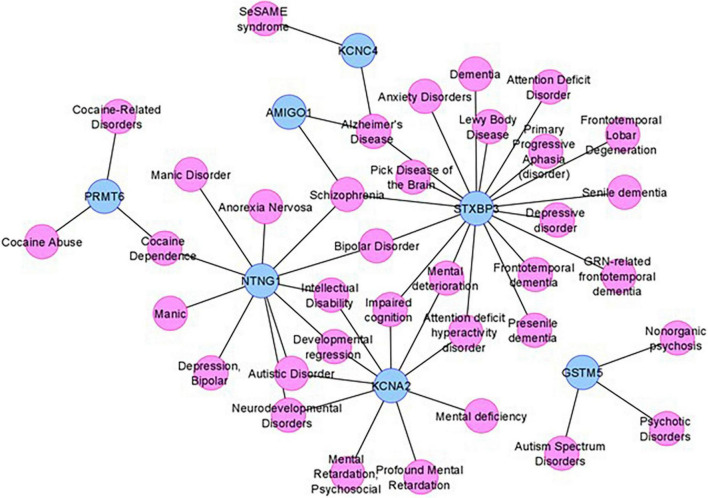
The mental disorder network. The candidate genes are depicted by blue nodes, and the disease terms are depicted by pink nodes. Edges correspond to disease associations.

To obtain additional evidence for our candidate genes being functionally involved in the clinical phenotype observed in our subjects, we consulted DisGeNET, one of the largest publicly available collections of genes and variants associated with human diseases (Pinero et al., 2020). After combining the lists of the five positional candidates (Table 2) and the 11 functional candidates (Supplementary Table 1), we focused on two specific disorders relevant to the phenotype studied here: “mental disorders” and “nervous system diseases.” Out of our 16 candidates, *FNDC7* is not present in DisGeNET, and seven are connected to at least one mental disorder confirming their involvement in the phenotype studied here (Figure 4). Among the 11 functional candidate genes, *STXBP3* and *KCNA2* seem to be particularly strong candidates since they are connected to disease terms like “impaired cognition” and “mental deterioration” among other relevant terms. The network for nervous system diseases is given in Supplementary Figure 1.

As DisGeNET was last updated in June 2020, we also consulted Human Phenotype Ontology (HPO), another resource for gene–disease relationships, which was last updated in June 2022. As in the results from DisGeNET, *KCNA2* is again linked to relevant terms like HP:0001268 (mental deterioration), HP:0001249 (intellectual disability), as well as HP:0000750 (delayed speech and language development), HP:0001263 (global developmental delay), and HP:0002317 (unsteady gait), additional relevant terms to the phenotype described in this article. *KCNA4* is also linked to HP:0000750 (delayed speech and language development) and HP:0002064 (spastic gait). Unfortunately, *STXBP3* could not be found in the HPO database nor any other genes given in Figure 4.

## Discussion

The CNVs represent a rich source of structural chromosomal variations that can result in severe phenotypic consequences as a result of gene dosage alteration, disruptions in coding sequences, and long-range gene regulators (Stranger et al., 2007). With the advancement of detection technology, it has become apparent that the collective involvement of CNVs underlies neurodevelopmental syndromes (Lee et al., 2015). Clinical features of seven heterozygous CNVs located at 1p13.3 have been described through karyotype–phenotype correlations (Tabata et al., 1991; Mattia et al., 1992; Lo et al., 1998; Utkus et al., 1999; Bisgaard et al., 2007; van Kuilenburg et al., 2009; Piccione et al., 2010) (Cases 1–7 in Table 1). Four of these published cases (Cases 4–7 in Table 1) are from the pre-microarray era, so the exact sizes, as well as the distal and proximal genomic breakpoints, of these large 1p13.3 deletions (9–21 Mb) are unknown.

Causative disease gene identification has been aided by refinement of critical regions through comparison of patients with overlapping large deletions like in Kallmann syndrome (Dode et al., 2003) and CHARGE syndrome (Vissers et al., 2004). However, understanding the genetic etiology of large pathogenic CNVs still remains a major challenge, because they frequently contain multiple genes (Rice and McLysaght, 2017).

By contrast, small CNVs containing a couple of genes provide a great potential in the identification of disease genes through comparative genomic mapping by aligning the genomic boundaries of CNVs with varying sizes and overlapping phenotypes. This was evidenced by an NDD candidate gene *SETD1B* identified from a critical 445 kb genomic region encompassing seven genes at 12q24.31 (Labonne et al., 2016). In the ensuing years, a large number of mutations in this gene were identified from next-generation sequencing (NGS) databases, verifying it as an NDD disease gene (Hiraide et al., 2018; Roston et al., 2021; Weerts et al., 2021).

Recently, we have recruited four new subjects having heterozygous microdeletions at 1p13.3 with common features of NDDs. To find out the candidate genes responsible for disease phenotypes, we aligned our cases with the aforementioned three published CNVs along with 22 informative DECIPHER cases on 1p13.3 as described in the “Results” section.

DECIPHER cases at 1p13.3 comprise 13 duplications, seven deletions, and two triplications (Supplementary Table 3). Nonetheless, most cases displayed neurodevelopmental phenotypes with various comorbidities. NDDs are linked to dosage alterations of several genes in both deletions and duplications (Rice and McLysaght, 2017). In addition, the majority of CNV cases are inherited from one parent with an unknown phenotype, while the inheritance of the remainders is unknown. This suggests that some CNVs at 1p13.3 might be inherited from an affected parent, have varying penetrance, or are imprinted epigenetically.

Our four subjects and seven patients reported in the literature share an overlapping phenotype of DD, ID, and facial dysmorphism (Table 1). This offered an opportunity for the identification of NDD genes that are responsible for a common set of phenotypes and features among all subjects with 1p13.3 interstitial deletions and duplications, regardless of whether they are inherited or *de novo.*

Based on our comparative *in silico* genomic mapping, we narrowed down the 4.6 Mb genomic region of 1p13.3 to four individual candidate gene loci and identified five positional candidate genes (*VAV3, WDR47, ELAPOR1, GSTM5*, and *LRIF1*) out of 59 genes in total (Figure 1A and Table 2). It is likely that the neurodevelopmental features in the majority of our CNV cases encompassing one or more of the five potential candidate genes are caused either by a single gene or by a combination of multiple genes among them.

Each gene has been interrogated in order to identify sporadic *de novo* variants in them or their interacting genes at the protein level in NDD subjects from the literature. Furthermore, their KO mice phenotype has been investigated to obtain supporting evidence (Table 2 and Supplementary Table 1), which has substantiated the candidacy of these positional candidate genes. We also confirmed the high expression of these genes in the brain and subsequently specific brain regions, suggesting their pathogenicity in NDDs (Figures 2, 3). The collective effort of *in silico* genome mapping informed by various disease gene databases, the role of interacting protein partners, and confirmation of gene expression in brain tissues strongly indicate the functional candidacy of these five positional genes in causing various NDDs.

Among the candidate genes we found, *VAV3* plays an important role in bone mass and remodeling, axon wiring in the ventrolateral medulla, and cerebellar development (Faccio et al., 2005). The cerebellum is known to be involved in motor learning and coordination. Consequentially, impairment of this region leads to dysfunctions in movement (Mapelli et al., 2022). This suggests a reason for the short stature, bone anomalies, and jerky gait pertaining to Subject 1 in this study (Table 1). VAV proteins have key signaling roles in the immune, cardiovascular, and nervous systems (Bustelo, 2014).

WDR47 belongs to the family of WD40-repeat (WDR) proteins. So far, mutations in 27 genes (9.4%) among 286 WDR genes have been linked to brain disorders, most notably ID associated with corpus callosum defects (Kannan et al., 2017). A smaller corpus callosum has been linked to an increased risk of autism (Paul et al., 2007), bipolar disorder (Li et al., 2014), and schizophrenia (Balevich et al., 2015). WDR47 is necessary for proper radial migration of projection neurons, and Wdr47 depletion compromises growth cone morphology and microtubule. The neuroanatomical defects found in Wdr47 KO result in hyperactivity and sensory motor gating abnormalities both in male and female mice (Kannan et al., 2017), suggesting a link between WDR47 and neurological disorder.

LRIF1 is required for accurate chromosome segregation in mitosis (Akram et al., 2018). A growing number of genes that regulate mitotic division are involved in neurodevelopmental diseases. Mitotic gene mutations give rise to insufficient cell proliferation and/or failure to replenish the neuronal stem cells in early development. The reduced number of neurons results in the underdevelopment of the central nervous system (CNS) and causes microcephaly and neuronal migration disorders (Degrassi et al., 2019). LRIF1 also interacts with many known genes involved in NDDs, which justifies its status as an NDD candidate gene (Table 2).

Another observation in the subjects and cases was that the severity of the phenotypes increases with the CNV size (Table 1 and Figure 1A). Three of the published cases (Cases 1–3 in Table 1) and Subject 1 with CNVs ranging from 3 to 14 Mb displayed severe cranial anomalies including microcephaly, macrocephaly, and asymmetry of the skull. However, among the remaining subjects with small CNVs, only Subject 4 displayed cranial anomalies that were considerably less pronounced than larger CNVs. Although Case 4 with the largest deletion did not show any apparent cranial anomalies, it remains to be seen whether she will develop secondary cranial anomalies after her infancy (Table 1). Larger deletions (Subject 1, Case 1, Case 2, Case 5, and Case 7) exhibited a distinct muscle disorder (mild fiber atrophy, diplegia, hypertonia, areflexia, and hypotonia) in comparison to Subjects 2–4 with small deletions, who did not show any muscle disorders (Table 1). Additionally, in contrast to Subjects 2–4 with small deletions, epilepsy was more pronounced in published cases with larger CNVs, including Subject 1, Case 4, and Case 6 (Table 1 and Figure 1A). Similarly, multiple hand, finger, feet, and toe anomalies were observed in all the published Cases 1–7 and Subject 1 that had significant large CNVs.

Furthermore, we also propose 11 additional genes, not encompassed within the demarcated loci, yet, pose as NDD candidate genes due to genetic variants in NDD subjects and KO mice with neurological phenotype. To support their candidacy, we also analyzed and compiled a list of interacting protein partners known to play a role in NDDs (Supplementary Table 1). These genes included in large CNVs (Subject 1 and Cases 1–3, Figure 1A and Table 1) were not contained in small CNVs, which were aligned to refine the candidate gene loci in *in silico* comparative genomic mapping. Hence, the clinical symptoms seen in these small CNVs might be caused by the position effect leading to altered expression of these 11 genes adjacent to them (Redon et al., 2005). Or these 11 genes might contribute to the phenotypes in the individuals with larger deletions and be irrelevant for cases with smaller CNVs.

Being the largest chromosome (249 Mb and representing 9% of the total human genome) containing a maximum number of 2,000 genes, chromosome 1 harbors the highest number of disease genes (Murphy et al., 2003).

We hypothesized that a total of 16 novel autosomal dominant candidate genes identified at 1p13.3 in this study are dosage sensitive in either direction and thus might not tolerate copy number change. These candidate genes will help zero in on NDD disease genes from NGS databases containing a large number of loss-of-function VUSs (variants of unknown significance) and autosomal dominant GUSs (genes of unknown significance).

## Clinical reports

### Subject 1 [46,XX,del(1)(p13.3), arr[hg19](chr1:107,240,429-110,671,860)x1]

Subject 1 (DGDP030) is a 44-year-old Asian woman, exhibiting a severe clinical phenotype as a result of a 3.4 Mb interstitial heterozygous deletion at 1p13.3. Her records indicate a normal birth, and her examination was within normal limits. She was adopted at 4 months of age; healthy appearing and quiet. She did not make eye contact or smile much; she would only cry when hungry and was unable to lie on her stomach. Yet, she was able to roam around the room on her back and she had a forceful grip. Her teeth did not appear until 20 months of age, after which, all grew within 3 months. Her mouth was too small for all her teeth and five had to be extracted. She then started to display mild hypotonia and suffered febrile seizures at 18 months of age.

At 3 years of age, she was diagnosed with the following symptoms: asymmetrical skull, mild anemia, febrile seizures, possible myoclonic seizures, hypotonia, hearing loss, and a recurring petechial rash. She was having seizures once a month. She also had strange night terror episodes and childhood schizophrenia. She was obsessive–compulsive and had wide mood swings that lasted 2–3 weeks each. The biopsy of her skeletal muscle from both gluteus and maximus at 18 years of age showed mild fiber atrophy and random variation in muscle fiber size. Her motor milestones were of poor quality and out of the usual order. She had a learning disability but could be directed to play; however, she preferred not to. She liked being in a room with people but did not make much of an effort to interact.

At the age of 31 years, she remained severely developmentally delayed and autistic. She had a jerky gait and would walk with arched feet. She constantly suffered from cramps in her legs and arms. She had an asymmetrical head with a smaller right parietal area, ptosis, astigmatism, low-set ears, unfolded ears, kyphosis, lordosis, and a high arched palate (Figure 1B). She held her hands at odd angles and held objects with her fingertips. At the age of 31 years, she had a complete emotional breakdown and increased violent behavior. At the same time, her tegretol levels were in the toxic range, and her thyroid levels were in the hypothyroid range.

At this age, she has had several bouts of myoglobinuria, unexplained fevers, recurrent infections, paradoxic and toxic reactions to medications, allergies to insect bites, hypothyroidism likely related to her seizure medication, thrombocytopenia due to medication, malignant hyperthermia, easily developed bruises, petechial rash, anemia, intermittent diarrhea, severe constipation, and both external canals intermittently plugged with cerumen. An auditory brainstem response evaluation was suggestive of a moderate peripheral deficit, potentially sensorineural, more so in the right ear than in the left ear. She was usually remarkably healthy with a normal echocardiogram and normal eye examination.

### Subject 2 [46,XY,del(1)(p13.3), arr[hg19](chr1:108,726,456-108,853,796)x1*dn*]

Subject 2 is an 11-year 6-month-old Caucasian boy with a history of global developmental delay, ID, and dysmorphic features. The child was born full term at 38 weeks of pregnancy and delivered by elective cesarean section from non-consanguineous parents. At birth, he weighed 2.650 kg (10*^th^* percentile) and his crown-heel length was 51 cm (50*^th^* percentile). Global developmental delay was observed at an early age. Up until the age of 11 years, no morphological phenotypic data were available for the child until specific genetic physicals revealed a bulbous nasal tip, epicanthal folds, downslanted palpebral fissure, low anterior hairline, long philtrum, wide nasal bridge, thin upper lip vermilion, long ears, micrognathia, and hypertelorism. At the age of 11 years, he weighed 32.5 kg (25*^th^* percentile) and measured 139 cm in height (25*^th^* percentile). He sat independently at around 12 months, walked at 24 months, spoke his first words at 36 months, and was not communicating in full sentences until 11 years of age. Now, at the age of 18 years, his speech has improved but he is not yet able to speak fluently. The MRI of the head showed no remarkable findings and remained likewise subsequently. Despite being recognized as an inattentive patient, psychological assessments revealed no attention deficit hyperactivity disorder (ADHD). However, he has shown severe learning difficulties, requiring individualized educational assistance to date. He has no significant anxiety disorder and shows continued progress; nevertheless, his deficits in attention and executive functions persist. Additional clinical laboratory tests have been carried out for this patient, including an extensive metabolic workup, chromosome analysis, and chromosomal microarray analysis. Except for the microarray result, all of the tests were uneventful. However, microarray indicated a *de novo* 130 kb heterozygous genomic loss at 1p13.3.

### Subject 3 [46,XX,del(1)(p13.3), arr[hg19](chr1:108,729,365-108,853,796)x1*dn*]

Subject 3 is a 6-year-old Caucasian girl with a history of neurodevelopmental delay, ID, epilepsy, and dysmorphic features. The child was born full term at 36 weeks of pregnancy and delivered by elective cesarean section. The biological parents stated that they could not rule out consanguinity; nonetheless, the child’s chromosomal microarray analysis did not show long continuous stretches of homozygosity. At birth, the newborn weighed 2.415 kg (5*^th^* percentile), and her crown-heel length was 50 cm (50*^th^* percentile). The Apgar scores were 07/09. As a newborn, the child showed no sucking reflex, requiring assistance to nursing. Moreover, at the age of 3 days old, she displayed jaundice. Up until the age of 6 years, no morphological phenotypic data were available for the child, when specific genetic physicals revealed epicanthal folds, downslanted palpebral fissure, long philtrum, wide nasal bridge, hypertelorism, and micrognathia. At the age of 6 years, the child had one seizure episode, and her MRI indicated a small arachnoid cyst in the posterior fossa. Global developmental delay was observed at an early age. She sat independently at around 10 months and walked at 16 months. Since birth, the child has shown loose skin. Additional clinical laboratory tests were performed including an extensive metabolic workup, chromosome analysis, and microarray analysis, which indicated a *de novo* 120 kb heterozygous genomic loss at 1p13.3.

### Subject 4 [46,XX,del(1)(p13.3), arr[hg19](chr1:109,878,638-110,200,728)x1*mat*]

Subject 4 is the first of four children of an unrelated Caucasian couple. The mother with the same microdeletion presents with global DD, behavioral disorders, and ID. Otherwise, the medical history of the father’s family was non-contributory. Vaginal delivery occurred at 39 weeks. At birth, growth parameters were within the normal range except for OFC < –2DS (birth weight 20*^th^* percentile/birth height 30*^th^* percentile). She walked at 18 months of age. She suffered from anxiety and intellectual disability, the reason why she was referred to a medical geneticist at the age of 15 years. On examination, she had a long face, epicanthus, prominent nose, posteriorly rotated ears, and short fingers. Her microcephaly was persistent. She had an array-CGH that revealed a maternally inherited 322 kb heterozygous deletion at 1p13.3 arr[hg19](chr1:109,878,638-110,200,728). Her oldest brother presents with global developmental delay, speech delay, and intellectual disability. Unfortunately, this family was lost to follow-up, and the segregation including the brother within the family could not get done. We were not able to get updated information regarding her development now that she is 22 years.

## Materials and methods

### Karyotype

For high-resolution karyotypes, peripheral blood lymphocytes from subjects were cultured with phytohematoagglutinin and harvested for cytogenetic analysis using standard techniques. Chromosome analysis was performed on GTL-banded chromosomes at an approximately 550 band level.

For Subjects 2 and 3, conventional cell cultures, harvesting, and GTG banding with a > 550 band resolution were performed following standard procedures. Chromosome analyses were done using Zeiss Axioscope^®^ (Göttingen, Germany) and the software IKAROS^®^ (Metasystems Corporation, Altlussheim, Germany).

For Subject 4, array-comparative genomic hybridization analysis (CGH) was performed using 180K Agilent microarray (Agilent Technologies, Santa Clara, CA, USA). Image analysis, normalization, and annotation were done with Feature Extraction 10.5.1.1 (Agilent, Santa Clara, USA) using the default settings. Data visualization and further analysis were performed with Cytogenomics 2.7.6.0 (Agilent, Santa Clara, USA). The array-CGH also detected a duplication of 17p11.2 of 194 kb (chr17:21307889-21502083), considered as benign.

### Genomic DNA extraction and microarray

The extraction of genomic DNA from Subject 1 was carried out using a standard phenol-chloroform protocol. To analyze submicroscopic copy number alterations in subjects, DNA samples from the subject’s blood and pooled normal controls (PROMEGA) were differentially labeled and cohybridized using a 44K whole-genome oligonucleotide array employing the protocols for array CGH provided by the manufacturer (Agilent, Santa Clara, USA). No deletion or duplication of other chromosomes was observed using the laboratory standard cutoff values (0.0Log2 ratio at a resolution of 0.3 Mb). Image analysis, normalization, and annotation were done with Feature Extraction 10.5.1.1 (Agilent, Santa Clara, USA) using the default settings. Data visualization and further analysis were performed with GenomeCAT^9^. CNVs were determined by circular binary segmentation.

Genomic DNAs from Subjects 2 and 3 as well as their parents were isolated from whole blood samples using the Illustra Blood Genomic Prep Mini Spin Kit (GE Healthcare Life Sciences, USA), following the manufacturer’s instructions. Chromosomal microarray analysis (CMA) was carried out on probands and their biological parents using the GeneChip^®^ CytoScanHD™ (ThermoFisher, USA) with 2.7 million polymorphic and non-polymorphic markers. Array analyses were done using the Chromosome Analysis Suite (ChAS^®^) software. The CNVs found in probands were analyzed in comparison with public databases, including the Database of Genomic Variants^10^ (DGV, version 107), the Database of Chromosomal Imbalance and Phenotype in Humans using Ensemble Resources (DECIPHER), and the CytoScan™ HD (High Density) Array Database.

### Fluorescence *in situ* hybridization

For Subject 1, BACs for probing genomic regions of interest were selected from the RPCI-11 (Roswell Park Cancer Institute) library *via* the UCSC genome browser^11^. Human genomic DNA inserts were extracted, fluorescently labeled using nick translation, and hybridized to a metaphase spread of the subject’s lymphocytes using standard procedures (Lichter et al., 1990).

### Comparative CNV mapping

The phenotypes and genomic coordinates from our four subjects (Subjects 1–4) were compared with three previously reported literature cases (Cases 1–3) (Bisgaard et al., 2007; van Kuilenburg et al., 2009; Piccione et al., 2010) and 22 unpublished DECIPHER CNVs with NDDs and less than 1 Mb in size (Figure 1A, Table 1, and Supplementary Table 3). Genomic coordinates from Case 1 were converted from hg17 to hg19 before the comparison was carried out. Furthermore, for the case from van Kuilenburg et al. (2009), the flanking distal and proximal genomic boundaries were between *TLCD4* and *WDR47* with the approximate genomic coordinates 95,558,073-109,584,850 (A.B.P. van Kuilenburg, personal communication, 19 May 2022) as reported in Figure 5 of their study.

### Quantitative reverse transcription PCR (RT-qPCR)

RT-qPCR was performed from the total RNA of the human brain and other tissues (Human Total RNA Master Panel II, Cat# 636643, Clontech). The catalog numbers of eight brain tissues from Clontech were as follows: dorsal root ganglion-636150, fetal brain total-636526, substantia nigra total-636560, cerebral cortex-636561, occipital lobe-636570, parietal lobe-636571, postcentral gyrus-636573, and hippocampus-636565. The cDNA synthesis was performed using 1–2 μg of total RNA using high-capacity cDNA reverse transcription kit and analyzed by RT-PCR on QuantStudio 6 Flex system using SYBR Green (ThermoFisher, Waltham, MA). We used ΔCt method to calculate the relative expression of each gene. In summary, relative gene expression was calculated by the difference between the Ct value (ΔCt) of the gene of interest and reference gene, GAPDH. After determining ΔCt, the fold change (2^–Δ*Ct*^) was measured, and the relative expression was plotted as excel graphs.

### Disease-gene network analysis

We performed disease-gene network analysis with DisGeNET (v7.0) (1) which contains 1,134,942 gene-disease associations between 21,671 genes and 30,170 diseases, disorders, traits, and clinical or abnormal human phenotypes. We used the DisGeNET Cytoscape app (Pinero et al., 2021) to browse and visualize the networks around the candidate genes. We used all sources, any association type and evidence level, and two specific disease classes of interest, namely, “mental disorders” and “nervous system diseases.”

## Data availability statement

The original contributions presented in this study are included in the article/Supplementary material, further inquiries can be directed to the corresponding author/s.

## Ethics statement

The studies involving human participants were reviewed and approved by Augusta University, Georgia, USA. Written informed consent to participate in this study was provided by the participants’ legal guardian/next of kin. Written informed consent was obtained from the minor(s)’ legal guardian/next of kin for the publication of any potentially identifiable images or data included in this article.

## Author contributions

AB-M and KJ contributed to the manuscript preparation including figure design and phenotypic data analysis. VG and PS performed the RT-qPCR and clinical examination of the patient data, respectively. ADLF performed the bioinformatics analysis. YP and KS collected and analyzed the data. CK, ADDC, ASDC, IP, LM, AS, LF, PC, and CR recruited the subjects and/or performed the clinical follow-up. LL, I-KK, C-HK, and W-YK edited the subsequent manuscript drafts and analyzed the data. H-GK conceived and designed the study, analyzed the data, and drafted the first manuscript. All authors read and approved the final manuscript.

## Footnotes

1
https://decipher.sanger.ac.uk/

2
https://my.qiagendigitalinsights.com/bbp/view/hgmd/pro/start.php

3
http://www.informatics.jax.org/

4
https://thebiogrid.org/

5
https://string-db.org/

6
https://www.disgenet.org/

7
https://hpo.jax.org/app

8
https://gtexportal.org/home/

9
http://www.molgen.mpg.de/$\sim $abt_rop/molecular_cytogenetics/CGHPRO.htmlwebcite

10
http://dgv.tcag.ca/dgv/app/home

11
http://genome.ucsc.edu

## Supplementary material

Supplementary Figure 1 The nervous system diseases network. The candidate genes are depicted by blue nodes and the disease terms by pink nodes. Edges correspond to disease associations. Out of our 16 candidates, 10 are connected to at least one nervous system disease providing more evidence for their involvement in the phenotype studied here.

## Abbreviations

CNV: copy number variation
NDDs: neurodevelopmental disorders
DD: development delay
ASD: autism spectrum disorder
ID: intellectual disability
CFA: craniofacial anomalies
ADHD: attention deficit hyperactivity disorder
NGS: next-generation sequencing.
