# Supplementary material for: A rigorous in silico genomic interrogation at 1p13.3 reveals 16 autosomal dominant candidate genes in syndromic neurodevelopmental disorders
Source: Front Mol Neurosci. 2022 Oct 6;15:979061. doi: 10.3389/fnmol.2022.979061 (PMC9582330; doi:10.3389/fnmol.2022.979061)
Supplement: Supplementary file 1 [file Table_1.docx]

| **No.** | **Gene candidate & MIM** | **Associated with a specific NDD phenotype in human or KO animal model** | **Protein interactors (references for interaction and variants in human with NDDs)** |
| --- | --- | --- | --- |
| 1 | PRMT6  (Protein arginine methyltransferase 6)  (608274) | 1. Neurodevelopmental disorder: de *novo* [NM_018137.3](https://www.ncbi.nlm.nih.gov/nucleotide/NM_018137.3): c.353A>C; [NP_060607.2](https://www.ncbi.nlm.nih.gov/protein/NP_060607.2): p.Q118P / de *novo* [NM_018137.3](https://www.ncbi.nlm.nih.gov/nucleotide/NM_018137.3): c.1079A>G; [NP_060607.2](https://www.ncbi.nlm.nih.gov/protein/NP_060607.2): p.K360R (Turner et al., 2019) 2. PRMTs were shown to have a role in the methylation of arginine-glycine rich amino acid residues in the C-terminal end of the fragile X mental retardation protein (FMRP). The study suggests that alterations in the activity of the PRMT during development hinders its ability to methylate FMRP, affecting its ability to bind RNA (Denman, 2002) 3. *PRMT8* might also be involved in neurodevelopmental disorders (Dong et al., 2021) 4. *PRMT9* (*FBXO11*) is associated with NDDs (Gregor et al., 2018) 5. A study carried out on zebrafish has elucidated an essential role for*PRMT6* for early development by directly suppressing stress sensor genes (Zhao et al., 2016) | 1. [***AKT3***](https://thebiogrid.org/115318/summary/homo-sapiens/akt3.html) **(Huttlin et al., 2017;Huttlin et al., 2021): microcephaly (Ballif et al., 2012), developmental delay, microcephaly, agenesis of corpus callosum, epilepsy, language delay and hearing impairment (Luo et al., 2018), intellectual disability (Gieldon et al., 2018), microcephaly, hypotonia, feeding difficulties, developmental delay and dysmorphic features (Gai et al., 2015)**      1. ***SNF8* (Weimann et al., 2013): autism spectrum disorder (Kim et al., 2020;Satterstrom et al., 2020), neurodevelopmental disorder (Turner et al., 2019)** 2. ***BMP3* (Weimann et al., 2013): autism spectrum disorder (Lim et al., 2017), autism (Turner et al., 2019), developmental delay (Coe et al., 2014), neurodevelopmental disorder (Turner et al., 2019)** |
| 2 | NTNG1  (Netrin G1)  (608818) | 1. Autism: de novo [NM_014917.4](https://www.ncbi.nlm.nih.gov/nucleotide/NM_014917.4): c.68A>G; [NP_055732.2](https://www.ncbi.nlm.nih.gov/protein/NP_055732.2): p.Y23C (Iossifov et al., 2014;Turner et al., 2019) / de novo [NM_014917.4](https://www.ncbi.nlm.nih.gov/nucleotide/NM_014917.4): c.404C>T; [NP_055732.2](https://www.ncbi.nlm.nih.gov/protein/NP_055732.2): p.T135I (O'Roak et al., 2012;Iossifov et al., 2014;Lim et al., 2017;Turner et al., 2019;Koire et al., 2021) 2. Intellectual disability, short stature and colobomata: Deletion of NTNG1 was suggested to cause intellectual disability (Bisgaard et al., 2007;van Kuilenburg et al., 2009) 3. Rett syndrome: disruption of NTNG1 by a balanced chromosome translocation in a girl with t(1;7)(p13.3;q31.3) and Rett syndrome (Borg et al., 2005) 4. NTNG1 may play an important role in the genesis of schizophrenia (Aoki-Suzuki et al., 2005;Zakharyan et al., 2011;Zhu et al., 2011;Wilcox and Quadri, 2014) 5. NGNT1 is expressed in the brain, especially strongly in the thalamus (Yin et al., 2002) 6. *NTNG1* plays a role in the laminar organization of dendrites and axonal guidance (Nishimura-Akiyoshi et al., 2007) 7. In mice models, knocking down Ntng1 in different neuronal subtypes is linked to changes in fear and anxiety-like behaviors (Zhang et al., 2016) | 1. [ADSL](https://thebiogrid.org/106667/summary/homo-sapiens/adsl.html) (Huttlin et al., 2021): autism (Turner et al., 2019), developmental disorder with epilepsy and malformations (Dong et al., 2020), intellectual disability / autism (Stone et al., 1992) 2. [ARIH1](https://thebiogrid.org/117348/summary/homo-sapiens/arih1.html) (Huttlin et al., 2021): neurodevelopmental disorder (Turner et al., 2019), neurodevelopmental disorder (Stessman et al., 2017), intellectual disability (Kosmicki et al., 2017) 3. [ARL8A](https://thebiogrid.org/126084/summary/homo-sapiens/arl8a.html) (Huttlin et al., 2021): global developmental delay (Di Gregorio et al., 2017) |
| 3 | FNDC7  **(Fibronectin type III domain containing 7)** | 1. Neurodevelopmental disorder: de novo [NM_001144937.3](https://www.ncbi.nlm.nih.gov/nucleotide/NM_001144937.3): c.243G>A; [NP_001138409.1](https://www.ncbi.nlm.nih.gov/protein/NP_001138409.1): p.T81= (Turner et al., 2019)/ de novo [NM_001144937.3](https://www.ncbi.nlm.nih.gov/nucleotide/NM_001144937.3): c.1451_1453delATG; [NP_001138409.1](https://www.ncbi.nlm.nih.gov/protein/NP_001138409.1): p.(Asp484del) (Deciphering Developmental Disorders, 2017;Turner et al., 2019) 2. Developmental and epileptic encephalopathy: [NM_001144937.3](https://www.ncbi.nlm.nih.gov/nucleotide/NM_001144937.3): c.1052T>G; [NP_001138409.1](https://www.ncbi.nlm.nih.gov/protein/NP_001138409.1): p.V351G (Takata et al., 2019) | 1. [DDX39A](https://thebiogrid.org/115507/summary/homo-sapiens/ddx39a.html) (Shi et al., 2020): autism (Turner et al., 2019) 2. [SNIP1](https://thebiogrid.org/122864/summary/homo-sapiens/snip1.html) (Chen et al., 2018): symptomatic epilepsy and skull dysplasia (Puffenberger et al., 2012;Capalbo et al., 2019) |
| 4 | STXBP3  (Syntaxin-binding protein 3)  (608339) | 1. Autism: de novo [NM_007269.4](https://www.ncbi.nlm.nih.gov/nucleotide/NM_007269.4): c.709A>G; [NP_009200.2](https://www.ncbi.nlm.nih.gov/protein/NP_009200.2): p.I237V (Turner et al., 2019) 2. Autism spectrum disorder: de novo [NM_007269.4](https://www.ncbi.nlm.nih.gov/nucleotide/NM_007269.4): c.709A>G; [NP_009200.2](https://www.ncbi.nlm.nih.gov/protein/NP_009200.2): p.I237V (Lim et al., 2017) / de novo [NM_007269.4](https://www.ncbi.nlm.nih.gov/nucleotide/NM_007269.4): c.593G>A; [NP_009200.2](https://www.ncbi.nlm.nih.gov/protein/NP_009200.2): p.S198N (Lim et al., 2017) 3. Neurodevelopmental disorder: de novo [NM_007269.4](https://www.ncbi.nlm.nih.gov/nucleotide/NM_007269.4): c.51_52delGA; [NP_009200.2](https://www.ncbi.nlm.nih.gov/protein/NP_009200.2): p.(Lys17Asnfs*7) (Turner et al., 2019) 4. In mice, loss of the gene encoding the widely expressed isoform Munc18c (which is other name of Stxbp3) induces intrauterine growth retardation and brain structural disruption (Tellam et al., 1995;Kanda et al., 2005) | 1. [AAGAB](https://thebiogrid.org/122835/summary/homo-sapiens/aagab.html) (Huttlin et al., 2021): autism (Turner et al., 2019), neurodevelopmental disorder (Deciphering Developmental Disorders, 2017;Turner et al., 2019) 2. [TUBA3C](https://thebiogrid.org/113129/summary/homo-sapiens/tuba3c.html) (Huttlin et al., 2021): autism (Turner et al., 2019), autism spectrum disorder (Lim et al., 2017) 3. [DIS3](https://thebiogrid.org/116559/summary/homo-sapiens/dis3.html) (Havugimana et al., 2012): autism spectrum disorder (Lim et al., 2017;Turner et al., 2019) |
| 5 | *CELSR2*  (Cadherin EGF lag seven-pass G-type receptor 2)  (604265) | 1. Autism: *de novo* [NM_001408.3](https://www.ncbi.nlm.nih.gov/nucleotide/NM_001408.3): c.6518G>A; [NP_001399.1](https://www.ncbi.nlm.nih.gov/protein/NP_001399.1): p.R2173H / de novo [NM_001408.3](https://www.ncbi.nlm.nih.gov/nucleotide/NM_001408.3): c.7701C>T; [NP_001399.1](https://www.ncbi.nlm.nih.gov/protein/NP_001399.1): p.A2567= (Turner et al., 2019) 2. Autism spectrum disorder : *de novo* [NM_001408.3](https://www.ncbi.nlm.nih.gov/nucleotide/NM_001408.3): c.8185G>C; [NP_001399.1](https://www.ncbi.nlm.nih.gov/protein/NP_001399.1): p.D2729H (Al-Mubarak et al., 2017) 3. Developmental delay and intellectual disability: autosomal recessive (Karaca et al., 2015) 4. Neurodevelopmental disorder: *de novo* [NM_001408.3](https://www.ncbi.nlm.nih.gov/nucleotide/NM_001408.3): c.1733T>G; [NP_001399.1](https://www.ncbi.nlm.nih.gov/protein/NP_001399.1): p.F578C (Turner et al., 2019) 5. Schizophrenia: *de novo* [NM_001408.3](https://www.ncbi.nlm.nih.gov/nucleotide/NM_001408.3): c.1268C>G; [NP_001399.1](https://www.ncbi.nlm.nih.gov/protein/NP_001399.1): p.A423G (Gulsuner et al., 2013) 6. Joubert syndrome with cortical heterotopia, microophthalmia, and growth hormone deficiency: compound heterozygous variants (NM_001408.2):c.1150G>A; p.Ala384Thr and (NM_001408.2):c.6908C>T; p.Thr2303Met (Vilboux et al., 2017) 7. Novel non-synonymous mutations detected in Chinese individuals with neural tube defects (NTDs) (Qiao et al., 2016) 8. In mouse, Celsr1-3 is widely expressed in the nervous system throughout development, from embryonic through early postnatal stages to adulthood. The brain and spinal cord highly express all three members (Hadjantonakis et al., 1997;Formstone et al., 2000;Tissir et al., 2002)   In mammals, RNAi-mediated Celsr2 knockdown leads to Purkinje neuron dendritic tree simplification and a reduction in cortical pyramidal neuron length (Shima et al., 2004) | 1. [*PCDH12*](https://thebiogrid.org/119445/summary/homo-sapiens/pcdh12.html) (Huttlin et al., 2017;Huttlin et al., 2021): cerebral palsy, intellectual disability & epilepsy (Suzuki-Muromoto et al., 2018)      1. *IL27RA* (Huttlin et al., 2017): autism spectrum disorder (Iossifov et al., 2014;Lim et al., 2017;Turner et al., 2019), developmental disorder (Deciphering Developmental Disorders, 2017)   [*TSPAN5*](https://thebiogrid.org/115405/summary/homo-sapiens/tspan5.html) (Huttlin et al., 2021): intellectual disability and epilepsy (Snoeijen-Schouwenaars et al., 2019) |
| 6 | AMIGO1  (Adhesion molecule with Ig-like domain 1)  (615689) | 1. Autism spectrum disorder: de novo [NM_020703.4](https://www.ncbi.nlm.nih.gov/nucleotide/NM_020703.4): c.1355G>T; [NP_065754.2](https://www.ncbi.nlm.nih.gov/protein/NP_065754.2): p.G452V (Kim et al., 2020) 2. Neurodevelopmental disorder: de novo [NM_020703.4](https://www.ncbi.nlm.nih.gov/nucleotide/NM_020703.4): c.826C>T; [NP_065754.2](https://www.ncbi.nlm.nih.gov/protein/NP_065754.2): p.R276C (Turner et al., 2019) 3. AMIGO1 was identified as candidate gene for human schizophrenia and similar psychiatric illnesses (Peltola et al., 2016) 4. AMIGO-1 colocalizes widely with both mammalian voltage-gated potassium channels Kv2.1 and Kv2.2 in neurons across the brain in a variety of mammalian species (Bishop et al., 2018) 5. Mice with a AMIGO deficiency show abnormal behavior, reduced KV2.1 protein levels, and alterations in neuronal electrophysiology (Peltola et al., 2016) 6. Mice deficient in AMIGO show several schizophrenia-related characteristics (Peltola et al., 2016) | 1. [PICK1](https://thebiogrid.org/114849/summary/homo-sapiens/pick1.html) (Huttlin et al., 2021): neurodevelopmental disorder (Turner et al., 2019) 2. [ADAM21](https://thebiogrid.org/114283/summary/homo-sapiens/adam21.html) (Huttlin et al., 2017;Huttlin et al., 2021): developmental and epileptic encephalopathy (Takata et al., 2019) 3. [B3GNT3](https://thebiogrid.org/115614/summary/homo-sapiens/b3gnt3.html) (Huttlin et al., 2017): autism spectrum disorder (Lim et al., 2017;Turner et al., 2019) |
| 7 | *EPS8L3*  (Eps8-like protein 3)  (614989) | 1. Developmental disorder: de novo [NM_139053.3](https://www.ncbi.nlm.nih.gov/nucleotide/NM_139053.3): c.97-60C>T (Deciphering Developmental Disorders, 2017) 2. Periventricular nodular heterotopia: de novo [NM_139053.3](https://www.ncbi.nlm.nih.gov/nucleotide/NM_139053.3): c.879G>T; [NP_620641.1](https://www.ncbi.nlm.nih.gov/protein/NP_620641.1): p.K293N (Heinzen et al., 2018) 3. Drosophila memory performance is well influenced by the expression levels of EPS8L3 (LaFerriere et al., 2011) | 1. ABI1 (Offenhauser et al., 2004): neurodevelopmental disorder (Jin et al., 2017;Edwards et al., 2020;Morton et al., 2021) 2. [SRPK2](https://thebiogrid.org/112611/summary/homo-sapiens/srpk2.html) (Varjosalo et al., 2013): autism spectrum disorder (An et al., 2014;Chen et al., 2017;Kosmicki et al., 2017;Lim et al., 2017;Turner et al., 2019), amyotrophic lateral sclerosis (Cooper-Knock et al., 2017) |
| 8 | STRIP1  (Striatin-interacting protein 1)  (617918) | 1. Developmental and epileptic encephalopathy: [NM_033088.4](https://www.ncbi.nlm.nih.gov/nucleotide/NM_033088.4): c.1376T>C; [NP_149079.2](https://www.ncbi.nlm.nih.gov/protein/NP_149079.2): p.L459P (Takata et al., 2019)/ [NM_033088.4](https://www.ncbi.nlm.nih.gov/nucleotide/NM_033088.4): c.2110G>A; [NP_149079.2](https://www.ncbi.nlm.nih.gov/protein/NP_149079.2): p.V704M (Takata et al., 2019) 2. Autism: de novo [NM_033088.4](https://www.ncbi.nlm.nih.gov/nucleotide/NM_033088.4): c.836delC; [NP_149079.2](https://www.ncbi.nlm.nih.gov/protein/NP_149079.2): p.(Pro279Leufs*5) (Turner et al., 2019) 3. Neurodevelopmental disorder : de novo [NM_033088.4](https://www.ncbi.nlm.nih.gov/nucleotide/NM_033088.4): c.1251T>C; [NP_149079.2](https://www.ncbi.nlm.nih.gov/protein/NP_149079.2): p.T417= (Turner et al., 2019) 4. Homozygous Strip1 (Strip1−/−) knockout is embryonic lethal implying that is playing a critical function in embryo development (Zhang et al., 2021) 5. STRIP1 is a protein with potential roles in neuronal development. It was first discovered as one among the striatin-interacting phosphatases and kinases (STRIPAK) complex's key components (Goudreault et al., 2009) | 1. [PPP2R1A](https://thebiogrid.org/111510/summary/homo-sapiens/ppp2r1a.html) (Huttlin et al., 2021): neurodevelopmental disorder (Lenaerts et al., 2021), developmental disorder (Deciphering Developmental Disorders, 2015), intellectual disability (Anazi et al., 2017), developmental and epileptic encephalopathy (Hamdan et al., 2017) 2. [STRN](https://thebiogrid.org/112674/summary/homo-sapiens/strn.html) (Huttlin et al., 2021): autism spectrum disorder (Kosmicki et al., 2017) 3. [PPP2CA](https://thebiogrid.org/111507/summary/homo-sapiens/ppp2ca.html) (Huttlin et al., 2021): neurodevelopmental disorder (Reynhout et al., 2019), intellectual disability (Reijnders et al., 2017;Reynhout et al., 2019) |
| 9 | KCNC4  (Potassium channel, voltage-gated, shaw-related subfamily, member 4)  (176265) | 1. Neurodevelopmental disorder: de novo [NM_001039574.3](https://www.ncbi.nlm.nih.gov/nucleotide/NM_001039574.3): c.1546dupC; [NP_001034663.1](https://www.ncbi.nlm.nih.gov/protein/NP_001034663.1): p.(Arg516Profs*7) (Deciphering Developmental Disorders, 2017;Turner et al., 2019) 2. Ataxia and dystonia: de novo [NM_001039574.3](https://www.ncbi.nlm.nih.gov/nucleotide/NM_001039574.3): c.23C>T; [NP_001034663.1](https://www.ncbi.nlm.nih.gov/protein/NP_001034663.1): p.S8F (Monies et al., 2017) 3. Bipolar disorder: de novo [NM_001039574.3](https://www.ncbi.nlm.nih.gov/nucleotide/NM_001039574.3): c.614G>A; [NP_001034663.1](https://www.ncbi.nlm.nih.gov/protein/NP_001034663.1): p.C205Y (Nishioka et al., 2021) 4. In the pilocarpine epileptic rat hippocampus, protein and transcript levels of Kv3.4 (Kcnc4 gene) channels were shown to be lower. Down-regulation of Kv3.4 in mossy fibers may contribute to increased presynaptic excitability, resulting in repeated seizures (Pacheco Otalora et al., 2011) | 1. [HSPA8](https://thebiogrid.org/109544/summary/homo-sapiens/hspa8.html) (Huttlin et al., 2017;Huttlin et al., 2021): Parkinson disease (Gialluisi et al., 2021), schizophrenia (Fromer et al., 2014;Kosmicki et al., 2017;Nishioka et al., 2021) 2. [NPLOC4](https://thebiogrid.org/120798/summary/homo-sapiens/nploc4.html) (Huttlin et al., 2017;Huttlin et al., 2021): neurodevelopmental disorder (Turner et al., 2019) 3. [AMFR](https://thebiogrid.org/106764/summary/homo-sapiens/amfr.html) (Huttlin et al., 2017;Huttlin et al., 2021): autism (Turner et al., 2019) |
| 10 | RBM15  (RNA-binding motif protein 15)  (606077) | 1. Autism spectrum disorder: de novo [NM_022768.5](https://www.ncbi.nlm.nih.gov/nucleotide/NM_022768.5): c.1593T>A; [NP_073605.4](https://www.ncbi.nlm.nih.gov/protein/NP_073605.4): p.H531Q (Lim et al., 2017) 2. Neurodevelopmental disorder: de novo [NM_022768.5](https://www.ncbi.nlm.nih.gov/nucleotide/NM_022768.5): c.2766C>T; [NP_073605.4](https://www.ncbi.nlm.nih.gov/protein/NP_073605.4): p.A922= (Turner et al., 2019) 3. Amyotrophic lateral sclerosis: de novo [NM_022768.5](https://www.ncbi.nlm.nih.gov/nucleotide/NM_022768.5): c.1787G>A; [NP_073605.4](https://www.ncbi.nlm.nih.gov/protein/NP_073605.4): p.R596H (Cooper-Knock et al., 2017) | 1. [CNOT4](https://thebiogrid.org/110912/summary/homo-sapiens/cnot4.html) (Zhang et al., 2015): autism (Iossifov et al., 2014), autism spectrum disorder (Satterstrom et al., 2020) 2. [BRD4](https://thebiogrid.org/117036/summary/homo-sapiens/brd4.html) (Shu et al., 2020): autism spectrum disorder (Lim et al., 2017;Turner et al., 2019) 3. [CTSB](https://thebiogrid.org/107888/summary/homo-sapiens/ctsb.html) (Liu et al., 2021): neurodevelopmental disorder (Deciphering Developmental Disorders, 2017;Turner et al., 2019), autism spectrum disorder (Satterstrom et al., 2020) |
| 11 | KCNA2  (Potassium channel, voltage-gated, shaker-related subfamily, member 2)  (176262) | Intellectual disability: autosomal recessive (Symonds et al., 2019;Riazuddin et al., 2020)  1. Developmental and epileptic encephalopathy: de novo [NM_004974.4](https://www.ncbi.nlm.nih.gov/nucleotide/NM_004974.4): c.889C>T; [NP_004965.1](https://www.ncbi.nlm.nih.gov/protein/NP_004965.1): p.R297W (Hamdan et al., 2017;Sachdev et al., 2017;Routier et al., 2019) 2. Ataxia & myoclonic epilepsy: de novo [NM_004974.4](https://www.ncbi.nlm.nih.gov/nucleotide/NM_004974.4): c.890G>A; [NP_004965.1](https://www.ncbi.nlm.nih.gov/protein/NP_004965.1): p.R297Q (Syrbe et al., 2015;Corbett et al., 2016;Masnada et al., 2017;Costain et al., 2019) 3. Epilepsy, mild language retardation, strabismus, mild ataxia, joint hyperlaxity & generalised hyperreflexia: de novo [NM_004974.4](https://www.ncbi.nlm.nih.gov/nucleotide/NM_004974.4): c.959C>T; [NP_004965.1](https://www.ncbi.nlm.nih.gov/protein/NP_004965.1): p.T320I (Fernandez-Marmiesse et al., 2019) 4. Rett-like syndrome with infantile onset seizures: : de novo [NM_004974.4](https://www.ncbi.nlm.nih.gov/nucleotide/NM_004974.4): c.1223T>C; [NP_004965.1](https://www.ncbi.nlm.nih.gov/protein/NP_004965.1): p.V408A (Allou et al., 2017) 5. Kcna2 codes for Kv1.2, the alpha subunit of a Shaker-like potassium channel with strong expression in the thalamocortic system of mammals. 6. The seizure susceptibility of Kcna2-null mice was drastically increased (Brew et al., 2007) 7. The majority of ko null mice kept their eyes open and had age-appropriate motor activities (walking, running, rearing, grooming, and exploring) (Brew et al., 2007) | 1. [***KCNA1***](https://thebiogrid.org/109942/summary/homo-sapiens/kcna4.html) **(Coleman et al., 1999): episodic ataxia (Imbrici et al., 2017), epileptic encephalopathy, infantile & cognitive impairment (Rogers et al., 2018),**  neurodevelopmental disorder **(Turner et al., 2019), episodic ataxia with cerebellar dysfunction & cognitive delay (Demos et al., 2009), intellectual disability (Gilissen et al., 2014), schizophrenia (Purcell et al., 2014), seizures, developmental delay & multiple joint contractures (Butler et al., 2017)** 2. [***KCNA4***](https://thebiogrid.org/109942/summary/homo-sapiens/kcna4.html) **(Coleman et al., 1999;Huttlin et al., 2021): abnormal striatum, congenital cataract and intellectual disability (Kaya et al., 2016;Kars et al., 2021), autism spectrum disorder (Lim et al., 2017)** 3. [***KCNA3***](https://thebiogrid.org/109942/summary/homo-sapiens/kcna4.html) **(Coleman et al., 1999;Huttlin et al., 2021):**  neurodevelopmental disorder **(Turner et al., 2019)** |

**Suppl. Table 1.** Eleven functional candidate genes at 1p13.3 were selected. The candidacy of these genes was substantiated by their sporadic variants reported, their physical interaction with known neurodevelopmental genes based on HGMD, BioGrid, STRING as well as KO animal phenotype. Due to the large number of interacting genes, only three interacting genes in NDD were described. KO animal data with neurobehavioral phenotype is also mentioned wherever available. Although *CELSR2* and *KCNA2* are involved in autosomal recessive ID, it is listed as candidate genes due to other NDD phenotypes in subjects with heterozygous variants.

**REFERENCES**

Al-Mubarak, B., Abouelhoda, M., Omar, A., AlDhalaan, H., Aldosari, M., Nester, M., . . . Al Tassan, N. (2017). Whole exome sequencing reveals inherited and de novo variants in autism spectrum disorder: a trio study from Saudi families. *Sci Rep, 7*(1), 5679. doi:10.1038/s41598-017-06033-1

Allou, L., Julia, S., Amsallem, D., El Chehadeh, S., Lambert, L., Thevenon, J., . . . Philippe, C. (2017). Rett-like phenotypes: expanding the genetic heterogeneity to the KCNA2 gene and first familial case of CDKL5-related disease. *Clin Genet, 91*(3), 431-440. doi:10.1111/cge.12784

An, J. Y., Cristino, A. S., Zhao, Q., Edson, J., Williams, S. M., Ravine, D., . . . Claudianos, C. (2014). Towards a molecular characterization of autism spectrum disorders: an exome sequencing and systems approach. *Transl Psychiatry, 4*, e394. doi:10.1038/tp.2014.38

Anazi, S., Maddirevula, S., Salpietro, V., Asi, Y. T., Alsahli, S., Alhashem, A., . . . Alkuraya, F. S. (2017). Expanding the genetic heterogeneity of intellectual disability. *Hum Genet, 136*(11-12), 1419-1429. doi:10.1007/s00439-017-1843-2

Aoki-Suzuki, M., Yamada, K., Meerabux, J., Iwayama-Shigeno, Y., Ohba, H., Iwamoto, K., . . . Yoshikawa, T. (2005). A family-based association study and gene expression analyses of netrin-G1 and -G2 genes in schizophrenia. *Biol Psychiatry, 57*(4), 382-393. doi:10.1016/j.biopsych.2004.11.022

Ballif, B. C., Rosenfeld, J. A., Traylor, R., Theisen, A., Bader, P. I., Ladda, R. L., . . . Shaffer, L. G. (2012). High-resolution array CGH defines critical regions and candidate genes for microcephaly, abnormalities of the corpus callosum, and seizure phenotypes in patients with microdeletions of 1q43q44. *Hum Genet, 131*(1), 145-156. doi:10.1007/s00439-011-1073-y

Bisgaard, A. M., Rasmussen, L. N., Moller, H. U., Kirchhoff, M., & Bryndorf, T. (2007). Interstitial deletion of the short arm of chromosome 1 (1p13.1p21.1) in a girl with mental retardation, short stature and colobomata. *Clin Dysmorphol, 16*(2), 109-112. doi:10.1097/01.mcd.0000228425.89660.bf

Bishop, H. I., Cobb, M. M., Kirmiz, M., Parajuli, L. K., Mandikian, D., Philp, A. M., . . . Trimmer, J. S. (2018). Kv2 Ion Channels Determine the Expression and Localization of the Associated AMIGO-1 Cell Adhesion Molecule in Adult Brain Neurons. *Front Mol Neurosci, 11*, 1. doi:10.3389/fnmol.2018.00001

Borg, I., Freude, K., Kubart, S., Hoffmann, K., Menzel, C., Laccone, F., . . . Kalscheuer, V. M. (2005). Disruption of Netrin G1 by a balanced chromosome translocation in a girl with Rett syndrome. *Eur J Hum Genet, 13*(8), 921-927. doi:10.1038/sj.ejhg.5201429

Brew, H. M., Gittelman, J. X., Silverstein, R. S., Hanks, T. D., Demas, V. P., Robinson, L. C., . . . Tempel, B. L. (2007). Seizures and reduced life span in mice lacking the potassium channel subunit Kv1.2, but hypoexcitability and enlarged Kv1 currents in auditory neurons. *J Neurophysiol, 98*(3), 1501-1525. doi:10.1152/jn.00640.2006

Butler, K. M., da Silva, C., Alexander, J. J., Hegde, M., & Escayg, A. (2017). Diagnostic Yield From 339 Epilepsy Patients Screened on a Clinical Gene Panel. *Pediatr Neurol, 77*, 61-66. doi:10.1016/j.pediatrneurol.2017.09.003

Capalbo, A., Valero, R. A., Jimenez-Almazan, J., Pardo, P. M., Fabiani, M., Jimenez, D., . . . Rodriguez, J. M. (2019). Optimizing clinical exome design and parallel gene-testing for recessive genetic conditions in preconception carrier screening: Translational research genomic data from 14,125 exomes. *PLoS Genet, 15*(10), e1008409. doi:10.1371/journal.pgen.1008409

Chen, L. L., Lin, H. P., Zhou, W. J., He, C. X., Zhang, Z. Y., Cheng, Z. L., . . . Guan, K. L. (2018). SNIP1 Recruits TET2 to Regulate c-MYC Target Genes and Cellular DNA Damage Response. *Cell Rep, 25*(6), 1485-1500 e1484. doi:10.1016/j.celrep.2018.10.028

Chen, Y., Bartanus, J., Liang, D., Zhu, H., Breman, A. M., Smith, J. L., . . . Yu, F. (2017). Characterization of chromosomal abnormalities in pregnancy losses reveals critical genes and loci for human early development. *Hum Mutat, 38*(6), 669-677. doi:10.1002/humu.23207

Coe, B. P., Witherspoon, K., Rosenfeld, J. A., van Bon, B. W., Vulto-van Silfhout, A. T., Bosco, P., . . . Eichler, E. E. (2014). Refining analyses of copy number variation identifies specific genes associated with developmental delay. *Nat Genet, 46*(10), 1063-1071. doi:10.1038/ng.3092

Coleman, S. K., Newcombe, J., Pryke, J., & Dolly, J. O. (1999). Subunit composition of Kv1 channels in human CNS. *J Neurochem, 73*(2), 849-858. doi:10.1046/j.1471-4159.1999.0730849.x

Cooper-Knock, J., Robins, H., Niedermoser, I., Wyles, M., Heath, P. R., Higginbottom, A., . . . Shaw, P. J. (2017). Targeted Genetic Screen in Amyotrophic Lateral Sclerosis Reveals Novel Genetic Variants with Synergistic Effect on Clinical Phenotype. *Front Mol Neurosci, 10*, 370. doi:10.3389/fnmol.2017.00370

Corbett, M. A., Bellows, S. T., Li, M., Carroll, R., Micallef, S., Carvill, G. L., . . . Gecz, J. (2016). Dominant KCNA2 mutation causes episodic ataxia and pharmacoresponsive epilepsy. *Neurology, 87*(19), 1975-1984. doi:10.1212/WNL.0000000000003309

Costain, G., Cordeiro, D., Matviychuk, D., & Mercimek-Andrews, S. (2019). Clinical Application of Targeted Next-Generation Sequencing Panels and Whole Exome Sequencing in Childhood Epilepsy. *Neuroscience, 418*, 291-310. doi:10.1016/j.neuroscience.2019.08.016

Deciphering Developmental Disorders, S. (2015). Large-scale discovery of novel genetic causes of developmental disorders. *Nature, 519*(7542), 223-228. doi:10.1038/nature14135

Deciphering Developmental Disorders, S. (2017). Prevalence and architecture of de novo mutations in developmental disorders. *Nature, 542*(7642), 433-438. doi:10.1038/nature21062

Demos, M. K., Macri, V., Farrell, K., Nelson, T. N., Chapman, K., Accili, E., & Armstrong, L. (2009). A novel KCNA1 mutation associated with global delay and persistent cerebellar dysfunction. *Mov Disord, 24*(5), 778-782. doi:10.1002/mds.22467

Denman, R. B. (2002). Methylation of the arginine-glycine-rich region in the fragile X mental retardation protein FMRP differentially affects RNA binding. *Cell Mol Biol Lett, 7*(3), 877-883. Retrieved from <https://www.ncbi.nlm.nih.gov/pubmed/12378270>

Di Gregorio, E., Riberi, E., Belligni, E. F., Biamino, E., Spielmann, M., Ala, U., . . . Ferrero, G. B. (2017). Copy number variants analysis in a cohort of isolated and syndromic developmental delay/intellectual disability reveals novel genomic disorders, position effects and candidate disease genes. *Clin Genet, 92*(4), 415-422. doi:10.1111/cge.13009

Dong, R., Li, X., & Lai, K. O. (2021). Activity and Function of the PRMT8 Protein Arginine Methyltransferase in Neurons. *Life (Basel), 11*(11). doi:10.3390/life11111132

Dong, X., Liu, B., Yang, L., Wang, H., Wu, B., Liu, R., . . . Lu, Y. (2020). Clinical exome sequencing as the first-tier test for diagnosing developmental disorders covering both CNV and SNV: a Chinese cohort. *J Med Genet, 57*(8), 558-566. doi:10.1136/jmedgenet-2019-106377

Edwards, J. J., Rouillard, A. D., Fernandez, N. F., Wang, Z., Lachmann, A., Shankaran, S. S., . . . Gelb, B. D. (2020). Systems Analysis Implicates WAVE2 Complex in the Pathogenesis of Developmental Left-Sided Obstructive Heart Defects. *JACC Basic Transl Sci, 5*(4), 376-386. doi:10.1016/j.jacbts.2020.01.012

Fernandez-Marmiesse, A., Roca, I., Diaz-Flores, F., Cantarin, V., Perez-Poyato, M. S., Fontalba, A., . . . Martinez-Atienza, M. (2019). Rare Variants in 48 Genes Account for 42% of Cases of Epilepsy With or Without Neurodevelopmental Delay in 246 Pediatric Patients. *Front Neurosci, 13*, 1135. doi:10.3389/fnins.2019.01135

Formstone, C. J., Barclay, J., Rees, M., & Little, P. F. (2000). Chromosomal localization of Celsr2 and Celsr3 in the mouse; Celsr3 is a candidate for the tippy (tip) lethal mutant on chromosome 9. *Mamm Genome, 11*(5), 392-394. doi:10.1007/s003350010073

Fromer, M., Pocklington, A. J., Kavanagh, D. H., Williams, H. J., Dwyer, S., Gormley, P., . . . O'Donovan, M. C. (2014). De novo mutations in schizophrenia implicate synaptic networks. *Nature, 506*(7487), 179-184. doi:10.1038/nature12929

Gai, D., Haan, E., Scholar, M., Nicholl, J., & Yu, S. (2015). Phenotypes of AKT3 deletion: a case report and literature review. *Am J Med Genet A, 167A*(1), 174-179. doi:10.1002/ajmg.a.36710

Gialluisi, A., Reccia, M. G., Modugno, N., Nutile, T., Lombardi, A., Di Giovannantonio, L. G., . . . Esposito, T. (2021). Identification of sixteen novel candidate genes for late onset Parkinson's disease. *Mol Neurodegener, 16*(1), 35. doi:10.1186/s13024-021-00455-2

Gieldon, L., Mackenroth, L., Kahlert, A. K., Lemke, J. R., Porrmann, J., Schallner, J., . . . Rump, A. (2018). Diagnostic value of partial exome sequencing in developmental disorders. *PLoS One, 13*(8), e0201041. doi:10.1371/journal.pone.0201041

Gilissen, C., Hehir-Kwa, J. Y., Thung, D. T., van de Vorst, M., van Bon, B. W., Willemsen, M. H., . . . Veltman, J. A. (2014). Genome sequencing identifies major causes of severe intellectual disability. *Nature, 511*(7509), 344-347. doi:10.1038/nature13394

Goudreault, M., D'Ambrosio, L. M., Kean, M. J., Mullin, M. J., Larsen, B. G., Sanchez, A., . . . Gingras, A. C. (2009). A PP2A phosphatase high density interaction network identifies a novel striatin-interacting phosphatase and kinase complex linked to the cerebral cavernous malformation 3 (CCM3) protein. *Mol Cell Proteomics, 8*(1), 157-171. doi:10.1074/mcp.M800266-MCP200

Gregor, A., Sadleir, L. G., Asadollahi, R., Azzarello-Burri, S., Battaglia, A., Ousager, L. B., . . . Zweier, C. (2018). De Novo Variants in the F-Box Protein FBXO11 in 20 Individuals with a Variable Neurodevelopmental Disorder. *Am J Hum Genet, 103*(2), 305-316. doi:10.1016/j.ajhg.2018.07.003

Gulsuner, S., Walsh, T., Watts, A. C., Lee, M. K., Thornton, A. M., Casadei, S., . . . McClellan, J. M. (2013). Spatial and temporal mapping of de novo mutations in schizophrenia to a fetal prefrontal cortical network. *Cell, 154*(3), 518-529. doi:10.1016/j.cell.2013.06.049

Hadjantonakis, A. K., Sheward, W. J., Harmar, A. J., de Galan, L., Hoovers, J. M., & Little, P. F. (1997). Celsr1, a neural-specific gene encoding an unusual seven-pass transmembrane receptor, maps to mouse chromosome 15 and human chromosome 22qter. *Genomics, 45*(1), 97-104. doi:10.1006/geno.1997.4892

Hamdan, F. F., Myers, C. T., Cossette, P., Lemay, P., Spiegelman, D., Laporte, A. D., . . . Michaud, J. L. (2017). High Rate of Recurrent De Novo Mutations in Developmental and Epileptic Encephalopathies. *Am J Hum Genet, 101*(5), 664-685. doi:10.1016/j.ajhg.2017.09.008

Havugimana, P. C., Hart, G. T., Nepusz, T., Yang, H., Turinsky, A. L., Li, Z., . . . Emili, A. (2012). A census of human soluble protein complexes. *Cell, 150*(5), 1068-1081. doi:10.1016/j.cell.2012.08.011

Heinzen, E. L., O'Neill, A. C., Zhu, X., Allen, A. S., Bahlo, M., Chelly, J., . . . Epilepsy Phenome/Genome, P. (2018). De novo and inherited private variants in MAP1B in periventricular nodular heterotopia. *PLoS Genet, 14*(5), e1007281. doi:10.1371/journal.pgen.1007281

Huttlin, E. L., Bruckner, R. J., Navarrete-Perea, J., Cannon, J. R., Baltier, K., Gebreab, F., . . . Gygi, S. P. (2021). Dual proteome-scale networks reveal cell-specific remodeling of the human interactome. *Cell, 184*(11), 3022-3040 e3028. doi:10.1016/j.cell.2021.04.011

Huttlin, E. L., Bruckner, R. J., Paulo, J. A., Cannon, J. R., Ting, L., Baltier, K., . . . Harper, J. W. (2017). Architecture of the human interactome defines protein communities and disease networks. *Nature, 545*(7655), 505-509. doi:10.1038/nature22366

Imbrici, P., Altamura, C., Gualandi, F., Mangiatordi, G. F., Neri, M., De Maria, G., . . . Desaphy, J. F. (2017). A novel KCNA1 mutation in a patient with paroxysmal ataxia, myokymia, painful contractures and metabolic dysfunctions. *Mol Cell Neurosci, 83*, 6-12. doi:10.1016/j.mcn.2017.06.006

Iossifov, I., O'Roak, B. J., Sanders, S. J., Ronemus, M., Krumm, N., Levy, D., . . . Wigler, M. (2014). The contribution of de novo coding mutations to autism spectrum disorder. *Nature, 515*(7526), 216-221. doi:10.1038/nature13908

Jin, S. C., Homsy, J., Zaidi, S., Lu, Q., Morton, S., DePalma, S. R., . . . Brueckner, M. (2017). Contribution of rare inherited and de novo variants in 2,871 congenital heart disease probands. *Nat Genet, 49*(11), 1593-1601. doi:10.1038/ng.3970

Kanda, H., Tamori, Y., Shinoda, H., Yoshikawa, M., Sakaue, M., Udagawa, J., . . . Kasuga, M. (2005). Adipocytes from Munc18c-null mice show increased sensitivity to insulin-stimulated GLUT4 externalization. *J Clin Invest, 115*(2), 291-301. doi:10.1172/JCI22681

Karaca, E., Harel, T., Pehlivan, D., Jhangiani, S. N., Gambin, T., Coban Akdemir, Z., . . . Lupski, J. R. (2015). Genes that Affect Brain Structure and Function Identified by Rare Variant Analyses of Mendelian Neurologic Disease. *Neuron, 88*(3), 499-513. doi:10.1016/j.neuron.2015.09.048

Kars, M. E., Basak, A. N., Onat, O. E., Bilguvar, K., Choi, J., Itan, Y., . . . Ozcelik, T. (2021). The genetic structure of the Turkish population reveals high levels of variation and admixture. *Proc Natl Acad Sci U S A, 118*(36). doi:10.1073/pnas.2026076118

Kaya, N., Alsagob, M., D'Adamo, M. C., Al-Bakheet, A., Hasan, S., Muccioli, M., . . . Al-Owain, M. (2016). KCNA4 deficiency leads to a syndrome of abnormal striatum, congenital cataract and intellectual disability. *J Med Genet, 53*(11), 786-792. doi:10.1136/jmedgenet-2015-103637

Kim, N., Kim, K. H., Lim, W. J., Kim, J., Kim, S. A., & Yoo, H. J. (2020). Whole Exome Sequencing Identifies Novel De Novo Variants Interacting with Six Gene Networks in Autism Spectrum Disorder. *Genes (Basel), 12*(1). doi:10.3390/genes12010001

Koire, A., Katsonis, P., Kim, Y. W., Buchovecky, C., Wilson, S. J., & Lichtarge, O. (2021). A method to delineate de novo missense variants across pathways prioritizes genes linked to autism. *Sci Transl Med, 13*(594). doi:10.1126/scitranslmed.abc1739

Kosmicki, J. A., Samocha, K. E., Howrigan, D. P., Sanders, S. J., Slowikowski, K., Lek, M., . . . Daly, M. J. (2017). Refining the role of de novo protein-truncating variants in neurodevelopmental disorders by using population reference samples. *Nat Genet, 49*(4), 504-510. doi:10.1038/ng.3789

LaFerriere, H., Ostrowski, D., Guarnieri, D. J., & Zars, T. (2011). The arouser EPS8L3 gene is critical for normal memory in Drosophila. *PLoS One, 6*(7), e22867. doi:10.1371/journal.pone.0022867

Lenaerts, L., Reynhout, S., Verbinnen, I., Laumonnier, F., Toutain, A., Bonnet-Brilhault, F., . . . Janssens, V. (2021). The broad phenotypic spectrum of PPP2R1A-related neurodevelopmental disorders correlates with the degree of biochemical dysfunction. *Genet Med, 23*(2), 352-362. doi:10.1038/s41436-020-00981-2

Lim, E. T., Uddin, M., De Rubeis, S., Chan, Y., Kamumbu, A. S., Zhang, X., . . . Walsh, C. A. (2017). Rates, distribution and implications of postzygotic mosaic mutations in autism spectrum disorder. *Nat Neurosci, 20*(9), 1217-1224. doi:10.1038/nn.4598

Liu, X., Huuskonen, S., Laitinen, T., Redchuk, T., Bogacheva, M., Salokas, K., . . . Varjosalo, M. (2021). SARS-CoV-2-host proteome interactions for antiviral drug discovery. *Mol Syst Biol, 17*(11), e10396. doi:10.15252/msb.202110396

Luo, A., Cheng, D., Yuan, S., Li, H., Du, J., Zhang, Y., . . . Tan, Y. Q. (2018). Maternal interchromosomal insertional translocation leading to 1q43-q44 deletion and duplication in two siblings. *Mol Cytogenet, 11*, 24. doi:10.1186/s13039-018-0371-7

Masnada, S., Hedrich, U. B. S., Gardella, E., Schubert, J., Kaiwar, C., Klee, E. W., . . . Rubboli, G. (2017). Clinical spectrum and genotype-phenotype associations of KCNA2-related encephalopathies. *Brain, 140*(9), 2337-2354. doi:10.1093/brain/awx184

Monies, D., Abouelhoda, M., AlSayed, M., Alhassnan, Z., Alotaibi, M., Kayyali, H., . . . Alkuraya, F. S. (2017). The landscape of genetic diseases in Saudi Arabia based on the first 1000 diagnostic panels and exomes. *Hum Genet, 136*(8), 921-939. doi:10.1007/s00439-017-1821-8

Morton, S. U., Shimamura, A., Newburger, P. E., Opotowsky, A. R., Quiat, D., Pereira, A. C., . . . Seidman, C. E. (2021). Association of Damaging Variants in Genes With Increased Cancer Risk Among Patients With Congenital Heart Disease. *JAMA Cardiol, 6*(4), 457-462. doi:10.1001/jamacardio.2020.4947

Nishimura-Akiyoshi, S., Niimi, K., Nakashiba, T., & Itohara, S. (2007). Axonal netrin-Gs transneuronally determine lamina-specific subdendritic segments. *Proc Natl Acad Sci U S A, 104*(37), 14801-14806. doi:10.1073/pnas.0706919104

Nishioka, M., Kazuno, A. A., Nakamura, T., Sakai, N., Hayama, T., Fujii, K., . . . Takata, A. (2021). Systematic analysis of exonic germline and postzygotic de novo mutations in bipolar disorder. *Nat Commun, 12*(1), 3750. doi:10.1038/s41467-021-23453-w

O'Roak, B. J., Vives, L., Girirajan, S., Karakoc, E., Krumm, N., Coe, B. P., . . . Eichler, E. E. (2012). Sporadic autism exomes reveal a highly interconnected protein network of de novo mutations. *Nature, 485*(7397), 246-250. doi:10.1038/nature10989

Offenhauser, N., Borgonovo, A., Disanza, A., Romano, P., Ponzanelli, I., Iannolo, G., . . . Scita, G. (2004). The eps8 family of proteins links growth factor stimulation to actin reorganization generating functional redundancy in the Ras/Rac pathway. *Mol Biol Cell, 15*(1), 91-98. doi:10.1091/mbc.e03-06-0427

Pacheco Otalora, L. F., Skinner, F., Oliveira, M. S., Farrell, B., Arshadmansab, M. F., Pandari, T., . . . Garrido-Sanabria, E. R. (2011). Chronic deficit in the expression of voltage-gated potassium channel Kv3.4 subunit in the hippocampus of pilocarpine-treated epileptic rats. *Brain Res, 1368*, 308-316. doi:10.1016/j.brainres.2010.10.047

Peltola, M. A., Kuja-Panula, J., Liuhanen, J., Voikar, V., Piepponen, P., Hiekkalinna, T., . . . Rauvala, H. (2016). AMIGO-Kv2.1 Potassium Channel Complex Is Associated With Schizophrenia-Related Phenotypes. *Schizophr Bull, 42*(1), 191-201. doi:10.1093/schbul/sbv105

Puffenberger, E. G., Jinks, R. N., Sougnez, C., Cibulskis, K., Willert, R. A., Achilly, N. P., . . . Strauss, K. A. (2012). Genetic mapping and exome sequencing identify variants associated with five novel diseases. *PLoS One, 7*(1), e28936. doi:10.1371/journal.pone.0028936

Purcell, S. M., Moran, J. L., Fromer, M., Ruderfer, D., Solovieff, N., Roussos, P., . . . Sklar, P. (2014). A polygenic burden of rare disruptive mutations in schizophrenia. *Nature, 506*(7487), 185-190. doi:10.1038/nature12975

Qiao, X., Liu, Y., Li, P., Chen, Z., Li, H., Yang, X., . . . Wang, H. (2016). Genetic analysis of rare coding mutations of CELSR1-3 in congenital heart and neural tube defects in Chinese people. *Clin Sci (Lond), 130*(24), 2329-2340. doi:10.1042/CS20160686

Reijnders, M. R. F., Ansor, N. M., Kousi, M., Yue, W. W., Tan, P. L., Clarkson, K., . . . Banka, S. (2017). RAC1 Missense Mutations in Developmental Disorders with Diverse Phenotypes. *Am J Hum Genet, 101*(3), 466-477. doi:10.1016/j.ajhg.2017.08.007

Reynhout, S., Jansen, S., Haesen, D., van Belle, S., de Munnik, S. A., Bongers, E., . . . Vissers, L. (2019). De Novo Mutations Affecting the Catalytic Calpha Subunit of PP2A, PPP2CA, Cause Syndromic Intellectual Disability Resembling Other PP2A-Related Neurodevelopmental Disorders. *Am J Hum Genet, 104*(1), 139-156. doi:10.1016/j.ajhg.2018.12.002

Riazuddin, S., Hussain, M., Razzaq, A., Iqbal, Z., Shahzad, M., Polla, D. L., . . . Riazuddin, S. (2020). Correction: Exome sequencing of Pakistani consanguineous families identifies 30 novel candidate genes for recessive intellectual disability. *Mol Psychiatry, 25*(11), 3101-3102. doi:10.1038/s41380-018-0128-z

Rogers, A., Golumbek, P., Cellini, E., Doccini, V., Guerrini, R., Wallgren-Pettersson, C., . . . Gurnett, C. A. (2018). De novo KCNA1 variants in the PVP motif cause infantile epileptic encephalopathy and cognitive impairment similar to recurrent KCNA2 variants. *Am J Med Genet A, 176*(8), 1748-1752. doi:10.1002/ajmg.a.38840

Routier, L., Verny, F., Barcia, G., Chemaly, N., Desguerre, I., Colleaux, L., & Nabbout, R. (2019). Exome sequencing findings in 27 patients with myoclonic-atonic epilepsy: Is there a major genetic factor? *Clin Genet, 96*(3), 254-260. doi:10.1111/cge.13581

Sachdev, M., Gainza-Lein, M., Tchapyjnikov, D., Jiang, Y. H., Loddenkemper, T., & Mikati, M. A. (2017). Novel clinical manifestations in patients with KCNA2 mutations. *Seizure, 51*, 74-76. doi:10.1016/j.seizure.2017.07.018

Satterstrom, F. K., Kosmicki, J. A., Wang, J., Breen, M. S., De Rubeis, S., An, J. Y., . . . Buxbaum, J. D. (2020). Large-Scale Exome Sequencing Study Implicates Both Developmental and Functional Changes in the Neurobiology of Autism. *Cell, 180*(3), 568-584 e523. doi:10.1016/j.cell.2019.12.036

Shi, P., Guo, Y., Su, Y., Zhu, M., Fu, Y., Chi, H., . . . Huang, J. (2020). SUMOylation of DDX39A Alters Binding and Export of Antiviral Transcripts to Control Innate Immunity. *J Immunol, 205*(1), 168-180. doi:10.4049/jimmunol.2000053

Shima, Y., Kengaku, M., Hirano, T., Takeichi, M., & Uemura, T. (2004). Regulation of dendritic maintenance and growth by a mammalian 7-pass transmembrane cadherin. *Dev Cell, 7*(2), 205-216. doi:10.1016/j.devcel.2004.07.007

Shu, S., Wu, H. J., Ge, J. Y., Zeid, R., Harris, I. S., Jovanovic, B., . . . Polyak, K. (2020). Synthetic Lethal and Resistance Interactions with BET Bromodomain Inhibitors in Triple-Negative Breast Cancer. *Mol Cell, 78*(6), 1096-1113 e1098. doi:10.1016/j.molcel.2020.04.027

Snoeijen-Schouwenaars, F. M., van Ool, J. S., Verhoeven, J. S., van Mierlo, P., Braakman, H. M. H., Smeets, E. E., . . . Willemsen, M. H. (2019). Diagnostic exome sequencing in 100 consecutive patients with both epilepsy and intellectual disability. *Epilepsia, 60*(1), 155-164. doi:10.1111/epi.14618

Stessman, H. A., Xiong, B., Coe, B. P., Wang, T., Hoekzema, K., Fenckova, M., . . . Eichler, E. E. (2017). Targeted sequencing identifies 91 neurodevelopmental-disorder risk genes with autism and developmental-disability biases. *Nat Genet, 49*(4), 515-526. doi:10.1038/ng.3792

Stone, R. L., Aimi, J., Barshop, B. A., Jaeken, J., Van den Berghe, G., Zalkin, H., & Dixon, J. E. (1992). A mutation in adenylosuccinate lyase associated with mental retardation and autistic features. *Nat Genet, 1*(1), 59-63. doi:10.1038/ng0492-59

Suzuki-Muromoto, S., Wakusawa, K., Miyabayashi, T., Sato, R., Okubo, Y., Endo, W., . . . Haginoya, K. (2018). A case of new PCDH12 gene variants presented as dyskinetic cerebral palsy with epilepsy. *J Hum Genet, 63*(6), 749-753. doi:10.1038/s10038-018-0432-0

Symonds, J. D., Zuberi, S. M., Stewart, K., McLellan, A., O'Regan, M., MacLeod, S., . . . Wilson, M. (2019). Incidence and phenotypes of childhood-onset genetic epilepsies: a prospective population-based national cohort. *Brain, 142*(8), 2303-2318. doi:10.1093/brain/awz195

Syrbe, S., Hedrich, U. B. S., Riesch, E., Djemie, T., Muller, S., Moller, R. S., . . . Lemke, J. R. (2015). De novo loss- or gain-of-function mutations in KCNA2 cause epileptic encephalopathy. *Nat Genet, 47*(4), 393-399. doi:10.1038/ng.3239

Takata, A., Nakashima, M., Saitsu, H., Mizuguchi, T., Mitsuhashi, S., Takahashi, Y., . . . Matsumoto, N. (2019). Comprehensive analysis of coding variants highlights genetic complexity in developmental and epileptic encephalopathy. *Nat Commun, 10*(1), 2506. doi:10.1038/s41467-019-10482-9

Tellam, J. T., McIntosh, S., & James, D. E. (1995). Molecular identification of two novel Munc-18 isoforms expressed in non-neuronal tissues. *J Biol Chem, 270*(11), 5857-5863. doi:10.1074/jbc.270.11.5857

Tissir, F., De-Backer, O., Goffinet, A. M., & Lambert de Rouvroit, C. (2002). Developmental expression profiles of Celsr (Flamingo) genes in the mouse. *Mech Dev, 112*(1-2), 157-160. doi:10.1016/s0925-4773(01)00623-2

Turner, T. N., Wilfert, A. B., Bakken, T. E., Bernier, R. A., Pepper, M. R., Zhang, Z., . . . Eichler, E. E. (2019). Sex-Based Analysis of De Novo Variants in Neurodevelopmental Disorders. *Am J Hum Genet, 105*(6), 1274-1285. doi:10.1016/j.ajhg.2019.11.003

van Kuilenburg, A. B., Meijer, J., Mul, A. N., Hennekam, R. C., Hoovers, J. M., de Die-Smulders, C. E., . . . Rubio-Gozalbo, M. E. (2009). Analysis of severely affected patients with dihydropyrimidine dehydrogenase deficiency reveals large intragenic rearrangements of DPYD and a de novo interstitial deletion del(1)(p13.3p21.3). *Hum Genet, 125*(5-6), 581-590. doi:10.1007/s00439-009-0653-6

Varjosalo, M., Keskitalo, S., Van Drogen, A., Nurkkala, H., Vichalkovski, A., Aebersold, R., & Gstaiger, M. (2013). The protein interaction landscape of the human CMGC kinase group. *Cell Rep, 3*(4), 1306-1320. doi:10.1016/j.celrep.2013.03.027

Vilboux, T., Malicdan, M. C., Roney, J. C., Cullinane, A. R., Stephen, J., Yildirimli, D., . . . Gunay-Aygun, M. (2017). CELSR2, encoding a planar cell polarity protein, is a putative gene in Joubert syndrome with cortical heterotopia, microophthalmia, and growth hormone deficiency. *Am J Med Genet A, 173*(3), 661-666. doi:10.1002/ajmg.a.38005

Weimann, M., Grossmann, A., Woodsmith, J., Ozkan, Z., Birth, P., Meierhofer, D., . . . Stelzl, U. (2013). A Y2H-seq approach defines the human protein methyltransferase interactome. *Nat Methods, 10*(4), 339-342. doi:10.1038/nmeth.2397

Wilcox, J. A., & Quadri, S. (2014). Replication of NTNG1 association in schizophrenia. *Psychiatr Genet, 24*(6), 266-268. doi:10.1097/YPG.0000000000000061

Yin, Y., Miner, J. H., & Sanes, J. R. (2002). Laminets: laminin- and netrin-related genes expressed in distinct neuronal subsets. *Mol Cell Neurosci, 19*(3), 344-358. doi:10.1006/mcne.2001.1089

Zakharyan, R., Boyajyan, A., Arakelyan, A., Gevorgyan, A., Mrazek, F., & Petrek, M. (2011). Functional variants of the genes involved in neurodevelopment and susceptibility to schizophrenia in an Armenian population. *Hum Immunol, 72*(9), 746-748. doi:10.1016/j.humimm.2011.05.018

Zhang, L., Tran, N. T., Su, H., Wang, R., Lu, Y., Tang, H., . . . Zhao, X. (2015). Cross-talk between PRMT1-mediated methylation and ubiquitylation on RBM15 controls RNA splicing. *Elife, 4*. doi:10.7554/eLife.07938

Zhang, Q., Sano, C., Masuda, A., Ando, R., Tanaka, M., & Itohara, S. (2016). Netrin-G1 regulates fear-like and anxiety-like behaviors in dissociable neural circuits. *Sci Rep, 6*, 28750. doi:10.1038/srep28750

Zhang, S., Dong, Y., Qiang, R., Zhang, Y., Zhang, X., Chen, Y., . . . Chai, R. (2021). Characterization of Strip1 Expression in Mouse Cochlear Hair Cells. *Front Genet, 12*, 625867. doi:10.3389/fgene.2021.625867

Zhao, X. X., Zhang, Y. B., Ni, P. L., Wu, Z. L., Yan, Y. C., & Li, Y. P. (2016). Protein Arginine Methyltransferase 6 (Prmt6) Is Essential for Early Zebrafish Development through the Direct Suppression of gadd45alphaa Stress Sensor Gene. *J Biol Chem, 291*(1), 402-412. doi:10.1074/jbc.M115.666347

Zhu, Y., Yang, H., Bi, Y., Zhang, Y., Zhen, C., Xie, S., . . . Liu, Y. (2011). Positive association between NTNG1 and schizophrenia in Chinese Han population. *J Genet, 90*(3), 499-502. doi:10.1007/s12041-011-0112-8
