# Supplementary material for: A rigorous in silico genomic interrogation at 1p13.3 reveals 16 autosomal dominant candidate genes in syndromic neurodevelopmental disorders
Source: Front Mol Neurosci. 2022 Oct 6;15:979061. doi: 10.3389/fnmol.2022.979061 (PMC9582330; doi:10.3389/fnmol.2022.979061)
Supplement: Supplementary file 2 [file Table_2.docx]

| **No.** | **Gene candidate & MIM** | **Variants found in patients with NDDs** |
| --- | --- | --- |
| 1 | SLC25A24 (608744) | Fontaine syndrome: *de novo* [NM_013386.5](https://www.ncbi.nlm.nih.gov/nucleotide/NM_013386.5): c.649C>T; [NP_037518.3](https://www.ncbi.nlm.nih.gov/protein/NP_037518.3): p.R217C (Ehmke et al., 2017;Writzl et al., 2017;Zhu et al., 2018)  1. Fontaine syndrome: de novo [NM_013386.5](https://www.ncbi.nlm.nih.gov/nucleotide/NM_013386.5): c.650G>A; [NP_037518.3](https://www.ncbi.nlm.nih.gov/protein/NP_037518.3): p.R217H (Ehmke et al., 2017;Writzl et al., 2017;Rodriguez-Garcia et al., 2018;Ryu et al., 2019) 2. Multiple congenital anomalies: de novo [NM_013386.5](https://www.ncbi.nlm.nih.gov/nucleotide/NM_013386.5): c.650G>A; [NP_037518.3](https://www.ncbi.nlm.nih.gov/protein/NP_037518.3): p.R217H (Bruel et al., 2019) |
| 2 | NBPF4 (613994) | No variants found in individuals with NDD phenotype |
| 3 | NBPF6 (613996) | No variants found in individuals with NDD phenotype |
| 4 | FAM102B | Autism: *de novo* [NM_001010883.3](https://www.ncbi.nlm.nih.gov/nucleotide/NM_001010883.3): c.1077A>G; [NP_001010883.2](https://www.ncbi.nlm.nih.gov/protein/NP_001010883.2): p.K359= (Turner et al., 2019) |
| 5 | HENMT1 (612178) | Schizophrenia: *de novo* [NM_144584.3](https://www.ncbi.nlm.nih.gov/nucleotide/NM_144584.3): c.253C>T; [NP_653185.2](https://www.ncbi.nlm.nih.gov/protein/NP_653185.2): p.R85* (Howrigan et al., 2020) / *de novo* [NM_144584.3](https://www.ncbi.nlm.nih.gov/nucleotide/NM_144584.3): c.508_509delCT; [NP_653185.2](https://www.ncbi.nlm.nih.gov/protein/NP_653185.2): p.(Leu170Valfs*12) (Howrigan et al., 2020) |
| 6 | AKNAD1 | Neurodevelopmental disorder: *de novo* [NM_152763.5](https://www.ncbi.nlm.nih.gov/nucleotide/NM_152763.5): c.1352C>T; [NP_689976.2](https://www.ncbi.nlm.nih.gov/protein/NP_689976.2): p.T451I (Turner et al., 2019) |
| 7 | SPATA42 | No variants found in individuals with NDD phenotype |
| 8 | GPSM2 (609245) | Chudley-McCullough syndrome: autosomal recessive [NM_013296.5](https://www.ncbi.nlm.nih.gov/nucleotide/NM_013296.5): c.1492C>T; [NP_037428.3](https://www.ncbi.nlm.nih.gov/protein/NP_037428.3): p.R498* (Hou et al., 2020)Neurodevelopmental disorder: *de novo* [NM_013296.5](https://www.ncbi.nlm.nih.gov/nucleotide/NM_013296.5): c.786G>A; [NP_037428.3](https://www.ncbi.nlm.nih.gov/protein/NP_037428.3): p.S262= (Turner et al., 2019) |
| 9 | *CLCC1* (617539) | 1. Autism: *de novo* [NM_015127.5](https://www.ncbi.nlm.nih.gov/nucleotide/NM_015127.5): c.301T>C; [NP_055942.1](https://www.ncbi.nlm.nih.gov/protein/NP_055942.1): p.L101= (Turner et al., 2019) |
| 10 | TAF13 (600774) | 1. Intellectual Disability and Microcephaly: autosomal recessive [NM_005645.4](https://www.ncbi.nlm.nih.gov/nucleotide/NM_005645.4): c.92T>A; [NP_005636.1](https://www.ncbi.nlm.nih.gov/protein/NP_005636.1): p.L31H (Tawamie et al., 2017) 2. Autism spectrum disorder: de novo [NM_005645.4](https://www.ncbi.nlm.nih.gov/nucleotide/NM_005645.4): c.110G>A; [NP_005636.1](https://www.ncbi.nlm.nih.gov/protein/NP_005636.1): p.R37Q (Guo et al., 2018) 3. Schizophrenia: de novo [NM_005645.4](https://www.ncbi.nlm.nih.gov/nucleotide/NM_005645.4): c.238C>T; [NP_005636.1](https://www.ncbi.nlm.nih.gov/protein/NP_005636.1): p.R80* (Fromer et al., 2014) / de novo [NM_005645.4](https://www.ncbi.nlm.nih.gov/nucleotide/NM_005645.4): c.85_88delAAGA; [NP_005636.1](https://www.ncbi.nlm.nih.gov/protein/NP_005636.1): p.(Lys29Aspfs*10) (Fromer et al., 2014) |
| 11 | TMEM167B | No variants found in individuals with NDD phenotype |
| 12 | *CFAP276* (618682) | 1. Charcot-Marie-Tooth disease: heterozygous missense mutations c.47A>T ([NM_001122961.3](https://www.ncbi.nlm.nih.gov/nucleotide/NM_001122961.3)); p.K16I ([NP_001116433.1](https://www.ncbi.nlm.nih.gov/protein/NP_001116433.1)) / c.329T>A ([NM_001122961.3](https://www.ncbi.nlm.nih.gov/nucleotide/NM_001122961.3)); p.I110N ([NP_001116433.1](https://www.ncbi.nlm.nih.gov/protein/NP_001116433.1)) (Sun et al., 2019) |
| 13 | SARS1 (607529) | 1. Neurodevelopmental disorder with microcephaly, ataxia, and seizures: autosomal recessive (Musante et al., 2017) |
| 14 | [PSRC1](https://my.qiagendigitalinsights.com/bbp/view/hgmd/pro/gene.php?gene=PSRC1) (613126) | 1. Autism spectrum disorder: de novo [NM_032636.8](https://www.ncbi.nlm.nih.gov/nucleotide/NM_032636.8): c.519+8G>C (Satterstrom et al., 2020) |
| 15 | [*MYBPHL*](https://my.qiagendigitalinsights.com/bbp/view/hgmd/pro/gene.php?gene=MYBPHL) | 1. Schizophrenia: *de novo*  [NM_001010985.3](https://www.ncbi.nlm.nih.gov/nucleotide/NM_001010985.3): c.235G>A; [NP_001010985.2](https://www.ncbi.nlm.nih.gov/protein/NP_001010985.2): p.G79S (Fromer et al., 2014) |
| 16 | SORT1 (602458) | 1. Neurodevelopmental disorder: de novo [NM_002959.7](https://www.ncbi.nlm.nih.gov/nucleotide/NM_002959.7): c.1217A>G; [NP_002950.3](https://www.ncbi.nlm.nih.gov/protein/NP_002950.3): p.N406S (Turner et al., 2019) |
| 17 | PSMA5 (176844) | No variants found in individuals with NDD phenotype |
| 18 | SYPL2 | 1. Autism : de novo [NM_001040709.2](https://www.ncbi.nlm.nih.gov/nucleotide/NM_001040709.2): c.171C>T; [NP_001035799.1](https://www.ncbi.nlm.nih.gov/protein/NP_001035799.1): p.S57=(Turner et al., 2019) |
| 19 | ATXN7L2 | 1. Autism : de novo [NM_153340.5](https://www.ncbi.nlm.nih.gov/nucleotide/NM_153340.5): c.2025G>A; [NP_699171.3](https://www.ncbi.nlm.nih.gov/protein/NP_699171.3): p.L675=(Turner et al., 2019) |
| 20 | *CYB561D1* | No variants found in individuals with NDD phenotype |
| 21 | [*GPR61*](https://my.qiagendigitalinsights.com/bbp/view/hgmd/pro/gene.php?gene=GPR61) (606916) | 1. Neurodevelopmental disorder: *de novo* [NM_031936.5](https://www.ncbi.nlm.nih.gov/nucleotide/NM_031936.5): c.1211A>G; [NP_114142.3](https://www.ncbi.nlm.nih.gov/protein/NP_114142.3): p.K404R (Turner et al., 2019) |
| 22 | *GNAI3* (139370) | 1. Auriculocondylar syndrome: novel variants associated with auriculocondylar syndrome strengthen a common dominant negative effect [NM_006496.4](https://www.ncbi.nlm.nih.gov/nucleotide/NM_006496.4): c.134G>T; [NP_006487.1](https://www.ncbi.nlm.nih.gov/protein/NP_006487.1): p.G45V (Rieder et al., 2012;Romanelli Tavares et al., 2015) |
| 23 | MIR197 (611189) | No variants found in individuals with NDD phenotype |
| 24 | GNAT2 (139340) | No variants found in individuals with NDD phenotype |
| 25 | AMPD2 (102771) | Pontocerebellar hypoplasia, type 9: autosomal recessive [NM_001368809.2](https://www.ncbi.nlm.nih.gov/nucleotide/NM_001368809.2): c.885C>A; [NP_001355738.1](https://www.ncbi.nlm.nih.gov/protein/NP_001355738.1): p.Y295* (Akizu et al., 2013)Spastic paraplegia: autosomal recessive [NM_001368809.2](https://www.ncbi.nlm.nih.gov/nucleotide/NM_001368809.2): c.157delT; [NP_001355738.1](https://www.ncbi.nlm.nih.gov/protein/NP_001355738.1): p.Cys53Alafs*80 (Novarino et al., 2014)Global developmental delay, epilepsy and brain malformation: autosomal recessive [NM_001368809.2](https://www.ncbi.nlm.nih.gov/nucleotide/NM_001368809.2): c.1457G>A; [NP_001355738.1](https://www.ncbi.nlm.nih.gov/protein/NP_001355738.1): p.R486Q (Maddirevula et al., 2020)Neurodevelopmental disorder: *de novo* [NM_001368809.2](https://www.ncbi.nlm.nih.gov/nucleotide/NM_001368809.2): c.1005T>C; [NP_001355738.1](https://www.ncbi.nlm.nih.gov/protein/NP_001355738.1): p.H335= / *de novo* [NM_001368809.2](https://www.ncbi.nlm.nih.gov/nucleotide/NM_001368809.2): c.1222A>C; [NP_001355738.1](https://www.ncbi.nlm.nih.gov/protein/NP_001355738.1): p.S408R (Turner et al., 2019)Autism spectrum disorder: *de novo* [NM_001368809.2](https://www.ncbi.nlm.nih.gov/nucleotide/NM_001368809.2): c.575G>A; [NP_001355738.1](https://www.ncbi.nlm.nih.gov/protein/NP_001355738.1): p.R192Q (Iossifov et al., 2014;Lim et al., 2017;Turner et al., 2019) |
| 26 | GSTM4 (138333) | No variants found in individuals with NDD phenotype |
| 27 | GSTM2 (138380) | No variants found in individuals with NDD phenotype |
| 28 | GSTM1 (138350) | No variants found in individuals with NDD phenotype |
| 29 | GSTM3 (138390) | Bipolar disorder: *de novo* [NM_000849.5](https://www.ncbi.nlm.nih.gov/nucleotide/NM_000849.5): c.298C>T; [NP_000840.2](https://www.ncbi.nlm.nih.gov/protein/NP_000840.2): p.R100* (Kataoka et al., 2016;Nishioka et al., 2021) |
| 30 | CSF1 (120420) | Autism spectrum disorder: *de novo* [NM_000757.6](https://www.ncbi.nlm.nih.gov/nucleotide/NM_000757.6): c.157C>T; [NP_000748.4](https://www.ncbi.nlm.nih.gov/protein/NP_000748.4): p.R53W (Kosmicki et al., 2017;Lim et al., 2017;Turner et al., 2019) / *de novo* [NM_000757.6](https://www.ncbi.nlm.nih.gov/nucleotide/NM_000757.6): c.406C>T; [NP_000748.4](https://www.ncbi.nlm.nih.gov/protein/NP_000748.4): p.R136* (Kosmicki et al., 2017) |
| 31 | *AHCYL1* (607826) | 1. Autism spectrum disorder: *de novo* [NM_006621.7](https://www.ncbi.nlm.nih.gov/nucleotide/NM_006621.7): c.214A>G; [NP_006612.2](https://www.ncbi.nlm.nih.gov/protein/NP_006612.2): p.T72A (Lim et al., 2017) |
| 32 | *ALX3* (606014) | 1. Frontorhiny: autosomal recessive [NM_006492.3](https://www.ncbi.nlm.nih.gov/nucleotide/NM_006492.3): c.502C>G; [NP_006483.2](https://www.ncbi.nlm.nih.gov/protein/NP_006483.2): p.L168V (Twigg et al., 2009) |
| 33 | *UBL4B* (611127) | No variants found in individuals with NDD phenotype |
| 34 | SLC6A17 (610299) | Intellectual disability with progressive tremor, speech impairment and behavioral problems: autosomal recessive [NM_001010898.4](https://www.ncbi.nlm.nih.gov/nucleotide/NM_001010898.4): c.484G>A; [NP_001010898.1](https://www.ncbi.nlm.nih.gov/protein/NP_001010898.1): p.G162R (Iqbal et al., 2015)  1. Autism spectrum disorder: de novo [NM_001010898.4](https://www.ncbi.nlm.nih.gov/nucleotide/NM_001010898.4): c.1303G>A; [NP_001010898.1](https://www.ncbi.nlm.nih.gov/protein/NP_001010898.1): p.V435M (Satterstrom et al., 2020) 2. Failure to thrive, global developmental delay and regression, motor delay and spasticity: autosomal recessive [NM_001010898.4](https://www.ncbi.nlm.nih.gov/nucleotide/NM_001010898.4): c.746C>T; [NP_001010898.1](https://www.ncbi.nlm.nih.gov/protein/NP_001010898.1): p.S249L (Monies et al., 2019) 3. Schizophrenia: de novo [NM_001010898.4](https://www.ncbi.nlm.nih.gov/nucleotide/NM_001010898.4): c.829G>A; [NP_001010898.1](https://www.ncbi.nlm.nih.gov/protein/NP_001010898.1): p.A277T (Fromer et al., 2014) |
| 35 | *SLC16A4* (603878) | Autism: *de novo* [NM_004696.3](https://www.ncbi.nlm.nih.gov/nucleotide/NM_004696.3): c.455G>A; [NP_004687.1](https://www.ncbi.nlm.nih.gov/protein/NP_004687.1): p.R152H (Turner et al., 2019) |
| 36 | *LAMTOR5* (608521) | Autism: *de novo* [NM_006402.3](https://www.ncbi.nlm.nih.gov/nucleotide/NM_006402.3): c.483C>T; [NP_006393.2](https://www.ncbi.nlm.nih.gov/protein/NP_006393.2): p.H161=(Turner et al., 2019) |
| 37 | *PROK1* (606233) | No variants found in individuals with NDD phenotype |
| 38 | *KCNA10* (602420) | No variants found in individuals with NDD phenotype |
| 39 | [*KCNA3*](https://my.qiagendigitalinsights.com/bbp/view/hgmd/pro/gene.php?gene=KCNA3) (176263) | Neurodevelopmental disorders: *de novo* [NM_002232.5](https://www.ncbi.nlm.nih.gov/nucleotide/NM_002232.5): c.234T>C; [NP_002223.3](https://www.ncbi.nlm.nih.gov/protein/NP_002223.3): p.C78=(Turner et al., 2019) |
| 40 | *CD53* (151525) | No variants found in individuals with NDD phenotype |
| 41 | *DRAM2* (613360) | No variants found in individuals with NDD phenotype |
| 42 | *CEPT1* (616751) | No variants found in individuals with NDD phenotype |
| 43 | *DENND2D* (615111) | No variants found in individuals with NDD phenotype |

**Suppl. Table 2**. 43 genes disqualified as autosomal dominant NDD candidate genes at 1p13.3 are listed in the order from the telomeric to centromeric region. 25 genes with genetic variants are not qualified due to their involvement in autosomal recessive NDD, non-NDD phenotype, or existence of only one single, two or synonymous variants . In remaining 18 genes-(NBPF4, NBPF6, SPATA42, TMEM167B, PSMA5, MIR197, CYB561D1, GNAT2, GSTM4, GSTM2, GSTM1, UBL4B, KCNA10, PROK1, CD53, DRAM2, CEPT1, DENND2D)-no variants were reported.

**REFERENCES**

Akizu, N., Cantagrel, V., Schroth, J., Cai, N., Vaux, K., Mccloskey, D., Naviaux, R.K., Van Vleet, J., Fenstermaker, A.G., Silhavy, J.L., Scheliga, J.S., Toyama, K., Morisaki, H., Sonmez, F.M., Celep, F., Oraby, A., Zaki, M.S., Al-Baradie, R., Faqeih, E.A., Saleh, M.A., Spencer, E., Rosti, R.O., Scott, E., Nickerson, E., Gabriel, S., Morisaki, T., Holmes, E.W., and Gleeson, J.G. (2013). AMPD2 regulates GTP synthesis and is mutated in a potentially treatable neurodegenerative brainstem disorder. *Cell* 154**,** 505-517.

Bruel, A.L., Nambot, S., Quere, V., Vitobello, A., Thevenon, J., Assoum, M., Moutton, S., Houcinat, N., Lehalle, D., Jean-Marcais, N., Orphanomix Physician's, G., Chevarin, M., Jouan, T., Poe, C., Callier, P., Tisserand, E., Philippe, C., Them, F.T.M., Duffourd, Y., Faivre, L., and Thauvin-Robinet, C. (2019). Increased diagnostic and new genes identification outcome using research reanalysis of singleton exome sequencing. *Eur J Hum Genet* 27**,** 1519-1531.

Ehmke, N., Graul-Neumann, L., Smorag, L., Koenig, R., Segebrecht, L., Magoulas, P., Scaglia, F., Kilic, E., Hennig, A.F., Adolphs, N., Saha, N., Fauler, B., Kalscheuer, V.M., Hennig, F., Altmuller, J., Netzer, C., Thiele, H., Nurnberg, P., Yigit, G., Jager, M., Hecht, J., Kruger, U., Mielke, T., Krawitz, P.M., Horn, D., Schuelke, M., Mundlos, S., Bacino, C.A., Bonnen, P.E., Wollnik, B., Fischer-Zirnsak, B., and Kornak, U. (2017). De Novo Mutations in SLC25A24 Cause a Craniosynostosis Syndrome with Hypertrichosis, Progeroid Appearance, and Mitochondrial Dysfunction. *Am J Hum Genet* 101**,** 833-843.

Fromer, M., Pocklington, A.J., Kavanagh, D.H., Williams, H.J., Dwyer, S., Gormley, P., Georgieva, L., Rees, E., Palta, P., Ruderfer, D.M., Carrera, N., Humphreys, I., Johnson, J.S., Roussos, P., Barker, D.D., Banks, E., Milanova, V., Grant, S.G., Hannon, E., Rose, S.A., Chambert, K., Mahajan, M., Scolnick, E.M., Moran, J.L., Kirov, G., Palotie, A., Mccarroll, S.A., Holmans, P., Sklar, P., Owen, M.J., Purcell, S.M., and O'donovan, M.C. (2014). De novo mutations in schizophrenia implicate synaptic networks. *Nature* 506**,** 179-184.

Guo, H., Wang, T., Wu, H., Long, M., Coe, B.P., Li, H., Xun, G., Ou, J., Chen, B., Duan, G., Bai, T., Zhao, N., Shen, Y., Li, Y., Wang, Y., Zhang, Y., Baker, C., Liu, Y., Pang, N., Huang, L., Han, L., Jia, X., Liu, C., Ni, H., Yang, X., Xia, L., Chen, J., Shen, L., Li, Y., Zhao, R., Zhao, W., Peng, J., Pan, Q., Long, Z., Su, W., Tan, J., Du, X., Ke, X., Yao, M., Hu, Z., Zou, X., Zhao, J., Bernier, R.A., Eichler, E.E., and Xia, K. (2018). Inherited and multiple de novo mutations in autism/developmental delay risk genes suggest a multifactorial model. *Mol Autism* 9**,** 64.

Hou, Y.C., Yu, H.C., Martin, R., Cirulli, E.T., Schenker-Ahmed, N.M., Hicks, M., Cohen, I.V., Jonsson, T.J., Heister, R., Napier, L., Swisher, C.L., Dominguez, S., Tang, H., Li, W., Perkins, B.A., Barea, J., Rybak, C., Smith, E., Duchicela, K., Doney, M., Brar, P., Hernandez, N., Kirkness, E.F., Kahn, A.M., Venter, J.C., Karow, D.S., and Caskey, C.T. (2020). Precision medicine integrating whole-genome sequencing, comprehensive metabolomics, and advanced imaging. *Proc Natl Acad Sci U S A* 117**,** 3053-3062.

Howrigan, D.P., Rose, S.A., Samocha, K.E., Fromer, M., Cerrato, F., Chen, W.J., Churchhouse, C., Chambert, K., Chandler, S.D., Daly, M.J., Dumont, A., Genovese, G., Hwu, H.G., Laird, N., Kosmicki, J.A., Moran, J.L., Roe, C., Singh, T., Wang, S.H., Faraone, S.V., Glatt, S.J., Mccarroll, S.A., Tsuang, M., and Neale, B.M. (2020). Exome sequencing in schizophrenia-affected parent-offspring trios reveals risk conferred by protein-coding de novo mutations. *Nat Neurosci* 23**,** 185-193.

Iossifov, I., O'roak, B.J., Sanders, S.J., Ronemus, M., Krumm, N., Levy, D., Stessman, H.A., Witherspoon, K.T., Vives, L., Patterson, K.E., Smith, J.D., Paeper, B., Nickerson, D.A., Dea, J., Dong, S., Gonzalez, L.E., Mandell, J.D., Mane, S.M., Murtha, M.T., Sullivan, C.A., Walker, M.F., Waqar, Z., Wei, L., Willsey, A.J., Yamrom, B., Lee, Y.H., Grabowska, E., Dalkic, E., Wang, Z., Marks, S., Andrews, P., Leotta, A., Kendall, J., Hakker, I., Rosenbaum, J., Ma, B., Rodgers, L., Troge, J., Narzisi, G., Yoon, S., Schatz, M.C., Ye, K., Mccombie, W.R., Shendure, J., Eichler, E.E., State, M.W., and Wigler, M. (2014). The contribution of de novo coding mutations to autism spectrum disorder. *Nature* 515**,** 216-221.

Iqbal, Z., Willemsen, M.H., Papon, M.A., Musante, L., Benevento, M., Hu, H., Venselaar, H., Wissink-Lindhout, W.M., Vulto-Van Silfhout, A.T., Vissers, L.E., De Brouwer, A.P., Marouillat, S., Wienker, T.F., Ropers, H.H., Kahrizi, K., Nadif Kasri, N., Najmabadi, H., Laumonnier, F., Kleefstra, T., and Van Bokhoven, H. (2015). Homozygous SLC6A17 mutations cause autosomal-recessive intellectual disability with progressive tremor, speech impairment, and behavioral problems. *Am J Hum Genet* 96**,** 386-396.

Kataoka, M., Matoba, N., Sawada, T., Kazuno, A.A., Ishiwata, M., Fujii, K., Matsuo, K., Takata, A., and Kato, T. (2016). Exome sequencing for bipolar disorder points to roles of de novo loss-of-function and protein-altering mutations. *Mol Psychiatry* 21**,** 885-893.

Kosmicki, J.A., Samocha, K.E., Howrigan, D.P., Sanders, S.J., Slowikowski, K., Lek, M., Karczewski, K.J., Cutler, D.J., Devlin, B., Roeder, K., Buxbaum, J.D., Neale, B.M., Macarthur, D.G., Wall, D.P., Robinson, E.B., and Daly, M.J. (2017). Refining the role of de novo protein-truncating variants in neurodevelopmental disorders by using population reference samples. *Nat Genet* 49**,** 504-510.

Lim, E.T., Uddin, M., De Rubeis, S., Chan, Y., Kamumbu, A.S., Zhang, X., D'gama, A.M., Kim, S.N., Hill, R.S., Goldberg, A.P., Poultney, C., Minshew, N.J., Kushima, I., Aleksic, B., Ozaki, N., Parellada, M., Arango, C., Penzol, M.J., Carracedo, A., Kolevzon, A., Hultman, C.M., Weiss, L.A., Fromer, M., Chiocchetti, A.G., Freitag, C.M., Autism Sequencing, C., Church, G.M., Scherer, S.W., Buxbaum, J.D., and Walsh, C.A. (2017). Rates, distribution and implications of postzygotic mosaic mutations in autism spectrum disorder. *Nat Neurosci* 20**,** 1217-1224.

Maddirevula, S., Kuwahara, H., Ewida, N., Shamseldin, H.E., Patel, N., Alzahrani, F., Alsheddi, T., Alobeid, E., Alenazi, M., Alsaif, H.S., Alqahtani, M., Alali, M., Al Ali, H., Helaby, R., Ibrahim, N., Abdulwahab, F., Hashem, M., Hanna, N., Monies, D., Derar, N., Alsagheir, A., Alhashem, A., Alsaleem, B., Alhebbi, H., Wali, S., Umarov, R., Gao, X., and Alkuraya, F.S. (2020). Analysis of transcript-deleterious variants in Mendelian disorders: implications for RNA-based diagnostics. *Genome Biol* 21**,** 145.

Monies, D., Abouelhoda, M., Assoum, M., Moghrabi, N., Rafiullah, R., Almontashiri, N., Alowain, M., Alzaidan, H., Alsayed, M., Subhani, S., Cupler, E., Faden, M., Alhashem, A., Qari, A., Chedrawi, A., Aldhalaan, H., Kurdi, W., Khan, S., Rahbeeni, Z., Alotaibi, M., Goljan, E., Elbardisy, H., Elkalioby, M., Shah, Z., Alruwaili, H., Jaafar, A., Albar, R., Akilan, A., Tayeb, H., Tahir, A., Fawzy, M., Nasr, M., Makki, S., Alfaifi, A., Akleh, H., Yamani, S., Bubshait, D., Mahnashi, M., Basha, T., Alsagheir, A., Khaled, M.A., Alsaleem, K., Almugbel, M., Badawi, M., Bashiri, F., Bohlega, S., Sulaiman, R., Tous, E., Ahmed, S., Algoufi, T., Al-Mousa, H., Alaki, E., Alhumaidi, S., Alghamdi, H., Alghamdi, M., Sahly, A., Nahrir, S., Al-Ahmari, A., Alkuraya, H., Almehaidib, A., Abanemai, M., Alsohaibaini, F., Alsaud, B., Arnaout, R., Abdel-Salam, G.M.H., Aldhekri, H., Alkhater, S., Alqadi, K., Alsabban, E., Alshareef, T., Awartani, K., Banjar, H., Alsahan, N., Abosoudah, I., Alashwal, A., Aldekhail, W., Alhajjar, S., Al-Mayouf, S., Alsemari, A., Alshuaibi, W., Altala, S., Altalhi, A., Baz, S., Hamad, M., Abalkhail, T., Alenazi, B., Alkaff, A., Almohareb, F., Al Mutairi, F., Alsaleh, M., Alsonbul, A., Alzelaye, S., Bahzad, S., Manee, A.B., Jarrad, O., Meriki, N., Albeirouti, B., Alqasmi, A., Albalwi, M., Makhseed, N., et al. (2019). Lessons Learned from Large-Scale, First-Tier Clinical Exome Sequencing in a Highly Consanguineous Population. *Am J Hum Genet* 105**,** 879.

Musante, L., Puttmann, L., Kahrizi, K., Garshasbi, M., Hu, H., Stehr, H., Lipkowitz, B., Otto, S., Jensen, L.R., Tzschach, A., Jamali, P., Wienker, T., Najmabadi, H., Ropers, H.H., and Kuss, A.W. (2017). Mutations of the aminoacyl-tRNA-synthetases SARS and WARS2 are implicated in the etiology of autosomal recessive intellectual disability. *Hum Mutat* 38**,** 621-636.

Nishioka, M., Kazuno, A.A., Nakamura, T., Sakai, N., Hayama, T., Fujii, K., Matsuo, K., Komori, A., Ishiwata, M., Watanabe, Y., Oka, T., Matoba, N., Kataoka, M., Alkanaq, A.N., Hamanaka, K., Tsuboi, T., Sengoku, T., Ogata, K., Iwata, N., Ikeda, M., Matsumoto, N., Kato, T., and Takata, A. (2021). Systematic analysis of exonic germline and postzygotic de novo mutations in bipolar disorder. *Nat Commun* 12**,** 3750.

Novarino, G., Fenstermaker, A.G., Zaki, M.S., Hofree, M., Silhavy, J.L., Heiberg, A.D., Abdellateef, M., Rosti, B., Scott, E., Mansour, L., Masri, A., Kayserili, H., Al-Aama, J.Y., Abdel-Salam, G.M.H., Karminejad, A., Kara, M., Kara, B., Bozorgmehri, B., Ben-Omran, T., Mojahedi, F., El Din Mahmoud, I.G., Bouslam, N., Bouhouche, A., Benomar, A., Hanein, S., Raymond, L., Forlani, S., Mascaro, M., Selim, L., Shehata, N., Al-Allawi, N., Bindu, P.S., Azam, M., Gunel, M., Caglayan, A., Bilguvar, K., Tolun, A., Issa, M.Y., Schroth, J., Spencer, E.G., Rosti, R.O., Akizu, N., Vaux, K.K., Johansen, A., Koh, A.A., Megahed, H., Durr, A., Brice, A., Stevanin, G., Gabriel, S.B., Ideker, T., and Gleeson, J.G. (2014). Exome sequencing links corticospinal motor neuron disease to common neurodegenerative disorders. *Science* 343**,** 506-511.

Rieder, M.J., Green, G.E., Park, S.S., Stamper, B.D., Gordon, C.T., Johnson, J.M., Cunniff, C.M., Smith, J.D., Emery, S.B., Lyonnet, S., Amiel, J., Holder, M., Heggie, A.A., Bamshad, M.J., Nickerson, D.A., Cox, T.C., Hing, A.V., Horst, J.A., and Cunningham, M.L. (2012). A human homeotic transformation resulting from mutations in PLCB4 and GNAI3 causes auriculocondylar syndrome. *Am J Hum Genet* 90**,** 907-914.

Rodriguez-Garcia, M.E., Cotrina-Vinagre, F.J., Cruz-Rojo, J., Garzon-Lorenzo, L., Carnicero-Rodriguez, P., Pozo, J.S., and Martinez-Azorin, F. (2018). A rare male patient with Fontaine progeroid syndrome caused by p.R217H de novo mutation in SLC25A24. *Am J Med Genet A* 176**,** 2479-2486.

Romanelli Tavares, V.L., Gordon, C.T., Zechi-Ceide, R.M., Kokitsu-Nakata, N.M., Voisin, N., Tan, T.Y., Heggie, A.A., Vendramini-Pittoli, S., Propst, E.J., Papsin, B.C., Torres, T.T., Buermans, H., Capelo, L.P., Den Dunnen, J.T., Guion-Almeida, M.L., Lyonnet, S., Amiel, J., and Passos-Bueno, M.R. (2015). Novel variants in GNAI3 associated with auriculocondylar syndrome strengthen a common dominant negative effect. *Eur J Hum Genet* 23**,** 481-485.

Ryu, J., Ko, J.M., and Shin, C.H. (2019). A 9-year-old Korean girl with Fontaine progeroid syndrome: a case report with further phenotypical delineation and description of clinical course during long-term follow-up. *BMC Med Genet* 20**,** 188.

Satterstrom, F.K., Kosmicki, J.A., Wang, J., Breen, M.S., De Rubeis, S., An, J.Y., Peng, M., Collins, R., Grove, J., Klei, L., Stevens, C., Reichert, J., Mulhern, M.S., Artomov, M., Gerges, S., Sheppard, B., Xu, X., Bhaduri, A., Norman, U., Brand, H., Schwartz, G., Nguyen, R., Guerrero, E.E., Dias, C., Autism Sequencing, C., I, P.-B.C., Betancur, C., Cook, E.H., Gallagher, L., Gill, M., Sutcliffe, J.S., Thurm, A., Zwick, M.E., Borglum, A.D., State, M.W., Cicek, A.E., Talkowski, M.E., Cutler, D.J., Devlin, B., Sanders, S.J., Roeder, K., Daly, M.J., and Buxbaum, J.D. (2020). Large-Scale Exome Sequencing Study Implicates Both Developmental and Functional Changes in the Neurobiology of Autism. *Cell* 180**,** 568-584 e523.

Sun, S.C., Ma, D., Li, M.Y., Zhang, R.X., Huang, C., Huang, H.J., Xie, Y.Z., Wang, Z.J., Liu, J., Cai, D.C., Liu, C.X., Yang, Q., Bao, F.X., Gong, X.L., Li, J.R., Hui, Z., Wei, X.F., Zhong, J.M., Zhou, W.J., Shang, X., Zhang, C., Liu, X.G., Tang, B.S., Xiong, F., and Xu, X.M. (2019). Mutations in C1orf194, encoding a calcium regulator, cause dominant Charcot-Marie-Tooth disease. *Brain* 142**,** 2215-2229.

Tawamie, H., Martianov, I., Wohlfahrt, N., Buchert, R., Mengus, G., Uebe, S., Janiri, L., Hirsch, F.W., Schumacher, J., Ferrazzi, F., Sticht, H., Reis, A., Davidson, I., Colombo, R., and Abou Jamra, R. (2017). Hypomorphic Pathogenic Variants in TAF13 Are Associated with Autosomal-Recessive Intellectual Disability and Microcephaly. *Am J Hum Genet* 100**,** 555-561.

Turner, T.N., Wilfert, A.B., Bakken, T.E., Bernier, R.A., Pepper, M.R., Zhang, Z., Torene, R.I., Retterer, K., and Eichler, E.E. (2019). Sex-Based Analysis of De Novo Variants in Neurodevelopmental Disorders. *Am J Hum Genet* 105**,** 1274-1285.

Twigg, S.R., Versnel, S.L., Nurnberg, G., Lees, M.M., Bhat, M., Hammond, P., Hennekam, R.C., Hoogeboom, A.J., Hurst, J.A., Johnson, D., Robinson, A.A., Scambler, P.J., Gerrelli, D., Nurnberg, P., Mathijssen, I.M., and Wilkie, A.O. (2009). Frontorhiny, a distinctive presentation of frontonasal dysplasia caused by recessive mutations in the ALX3 homeobox gene. *Am J Hum Genet* 84**,** 698-705.

Writzl, K., Maver, A., Kovacic, L., Martinez-Valero, P., Contreras, L., Satrustegui, J., Castori, M., Faivre, L., Lapunzina, P., Van Kuilenburg, A.B.P., Radovic, S., Thauvin-Robinet, C., Peterlin, B., Del Arco, A., and Hennekam, R.C. (2017). De Novo Mutations in SLC25A24 Cause a Disorder Characterized by Early Aging, Bone Dysplasia, Characteristic Face, and Early Demise. *Am J Hum Genet* 101**,** 844-855.

Zhu, N., Gonzaga-Jauregui, C., Welch, C.L., Ma, L., Qi, H., King, A.K., Krishnan, U., Rosenzweig, E.B., Ivy, D.D., Austin, E.D., Hamid, R., Nichols, W.C., Pauciulo, M.W., Lutz, K.A., Sawle, A., Reid, J.G., Overton, J.D., Baras, A., Dewey, F., Shen, Y., and Chung, W.K. (2018). Exome Sequencing in Children With Pulmonary Arterial Hypertension Demonstrates Differences Compared With Adults. *Circ Genom Precis Med* 11**,** e001887.
