# Supplementary material for: A rigorous in silico genomic interrogation at 1p13.3 reveals 16 autosomal dominant candidate genes in syndromic neurodevelopmental disorders
Source: Front Mol Neurosci. 2022 Oct 6;15:979061. doi: 10.3389/fnmol.2022.979061 (PMC9582330; doi:10.3389/fnmol.2022.979061)
Supplement: Supplementary file 3 [file Table_3.docx]

| **DECIPHER ID** | **Copy number Variation (CNV)** | **Size (Kb)** | **Genomic coordinates [hg19]** | **Inheritance** | **Phenotype** |
| --- | --- | --- | --- | --- | --- |
| DCP290849 | deletion | 62 | 111,495,549-111,557,954 | paternally inherited, father with unknown phenotype | behavioral abnormality; intellectual disability |
| DCP370073 | deletion | 86 | 110,178,180-110,264,600 | paternally inherited, father with unknown phenotype | intellectual disability; seizures |
| DCP366561 | triplication | 131 | 110,260,816-110,392,345 | unknown | behavioral abnormality |
| DCP288610 | deletion | 141 | 108,739,569-108,881,454 | unknown | orofacial dyskinesia; progressive choreoathetosis; progressive spastic quadriplegia; rigidity |
| DCP383993 | deletion | 186 | 108,713,464-108,900,263 | maternally inherited, mother with unknown phenotype | abnormality of the face; biparietal narrowing; cryptorchidism; decreased fetal movement; feeding difficulties; frontal bossing; intrauterine growth retardation; micropenis; muscular hypotonia; neonatal hypotonia; intellectual disability; scrotal hypoplasia; short foot; small hand; thin vermilion border |
| DCP272657 | duplication | 193 | 109,472,445-109,666,050 | inherited from parent with unknown phenotype | intellectual disability |
| DCP305835 | duplication | 200 | 109,512,869-109,713,615 | unknown | poor coordination |
| DCP277822 | duplication | 207 | 109,265,154-109,472,504 | paternally inherited, father with unknown phenotype | ataxia; intellectual disability; strabismus |
| DCP429087 | duplication | 317 | 107,744,249-108,061,823 | unknown | autism |
| DCP274287 | duplication | 372 | 108,352,308-108,724,614 | maternally inherited, mother with unknown phenotype | intellectual disability |
| DCP276282 | duplication | 372 | 108,352,308-108,724,614 | maternally inherited, mother with unknown phenotype | intellectual disability |
| DCP403656 | duplication | 410 | 108,331,922-108,742,315 | unknown | epicanthus; irregular phalanges; long foot; low-set ears; synophrys; wide mouth |
| DCP291797 | triplication | 414 | 109,351,734-109,766,687 | paternally inherited, father with unknown phenotype | abnormal ventricular septum morphology; atrial septal defect; delayed speech; language development; intellectual disability |
| DCP331116 | duplication | 422 | 108,317,399-108,739,628 | unknown | growth abnormality (tall stature) |
| DCP414583 | deletion | 504 | 108,464,499-108,968,593 | *de novo* | absent earlobe; cupped ear; hemifacial hypoplasia |
| DCP331558 | deletion | 508 | 109,512,744-110,021,158 | unknown | behavioral abnormality; intellectual disability |
| DCP332790 | duplication | 508 | 109,241,217-109,749,844 | maternally inherited, mother with unknown phenotype | autism; delayed speech and language development |
| DCP303013 | deletion | 519 | 108,990,189-109,509,580 | unknown | generalized neonatal hypotonia; narrow palate; intellectual disability; mild, long fingers; mild short stature; moderate global developmental delay; wide intermammillary distance |
| DCP255098 | duplication | 541 | 108,312,657-108,854,165 | unknown | ataxia; bilateral tonic-clonic seizure; blepharophimosis; cafe-au-lait spot; delayed speech and language development; intellectual disability; protruding ear; sandal gap; short philtrum; small face; strabismus |
| DCP249535 | duplication | 766 | 109,192,181-109,958,614 | unknown | abnormality of hair pigmentation; clubbing; echolalia; inguinal hernia; pes planus; short stature; spasticity |
| DCP283721 | duplication | 773 | 107,951,320-108,724,614 | paternally inherited, father with unknown phenotype | behavioral abnormality; global developmental delay |
| DCP339124 | duplication | 962 | 108,703,829-109,666,050 | unknown | intellectual disability |

**Suppl. Table 3.** Twenty-two DECIPHER CNV cases used for *in silico* comparative genomic mapping at 1p13.3 in Figure 1A and the neurodevelopmental phenotypes in the carriers.
