# Supplementary figures and images for: A rigorous in silico genomic interrogation at 1p13.3 reveals 16 autosomal dominant candidate genes in syndromic neurodevelopmental disorders
Source: Front Mol Neurosci. 2022 Oct 6;15:979061. doi: 10.3389/fnmol.2022.979061 (PMC9582330; doi:10.3389/fnmol.2022.979061)

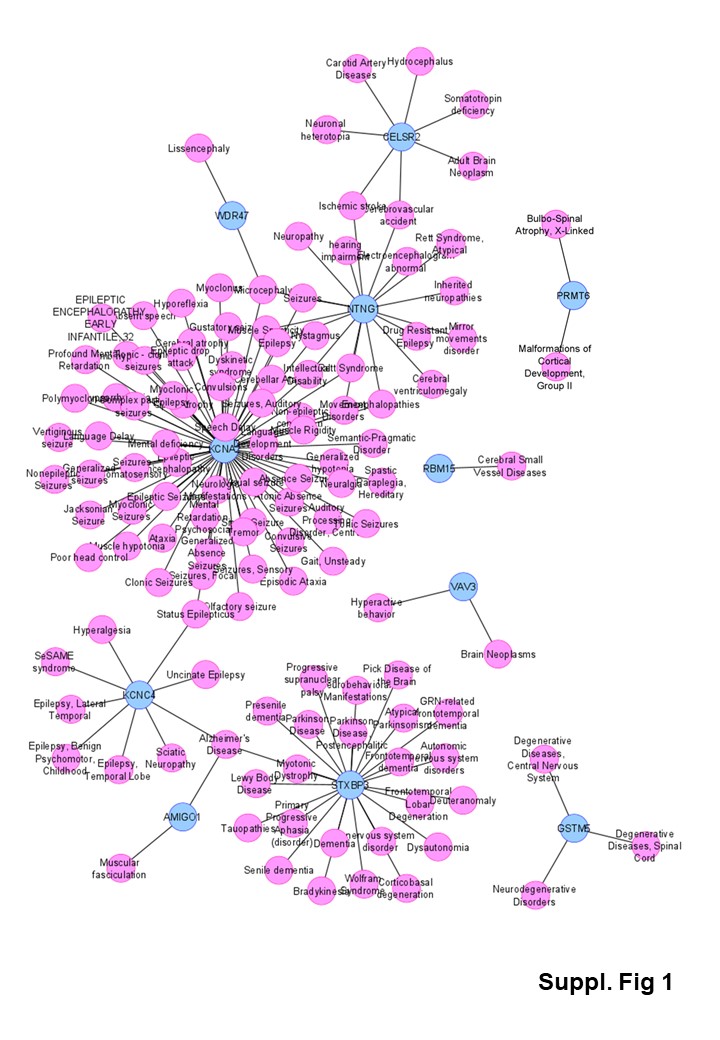

Supplement: Supplementary Figure 1 — The nervous system diseases network. The candidate genes are depicted by blue nodes and the disease terms by pink nodes. Edges correspond to disease associations. Out of our 16 candidates, 10 are connected to at least one nervous system disease providing more evidence for their involvement in the phenotype studied here. [file Image_1.JPEG]
